# Fabrication of mesoporous POMs/SiO_2_ nanofibers through electrospinning for oxidative conversion of biomass by H_2_O_2_ and oxygen†

**DOI:** 10.1039/c7ra12842h

**Published:** 2018-01-17

**Authors:** Siqi Yan, Yue Li, Peili Li, Ting Jia, Shengtian Wang, Xiaohong Wang

**Affiliations:** Key Lab of Polyoxometalate Science of Ministry of Education, Faculty of Chemistry, Northeast Normal University Changchun 130024 P. R. China wangxh665@nenu.edu.cn

## Abstract

Polyoxometalate H_5_PMo_10_V_2_O_40_/SiO_2_ (HPMoV/SiO_2_) nanofibers with a mesoporous structure were fabricated through combined electrospinning and surfactant-directing pore formation technique. These heterogeneous nanofibers were characterized by a wide variety of techniques to show mesoporous structure, high surface area, excellent stability, nanofiber morphology, highly efficient activity and selectivity towards the oxidation of starch and 5-hydroxymethylfurfural (5-HMF) either by H_2_O_2_ or by oxygen. POM/SiO_2_ nanofibers were suitable for oxidation of starch by H_2_O_2_ and oxidation of 5-HMF by O_2_ with high efficiency of 0.58 mol per 100 g carboxyl content and 89.2% yield of DFF; these results are comparable with those obtained using homogeneous H_5_PMo_10_V_2_O_40_ and HPMoV/SiO_2_ without nanofiber morphology. Furthermore, HPMoV/SiO_2_ nanofibers could be easily recycled and reused at least ten times with no significant loss of catalytic activity due to their nanofiber morphology.

## Abstract 2

The oxidation process for mesoporous H_5_PMo_10_V_2_O_40_/SiO_2_ nanofiber catalyst.

## Graphical abstract

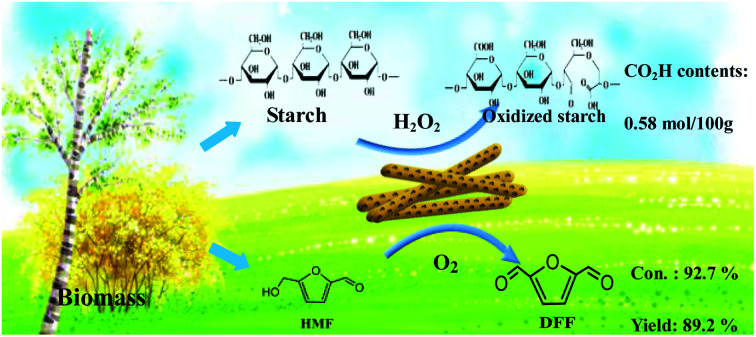

## 1. Introduction

**Scheme 1:**
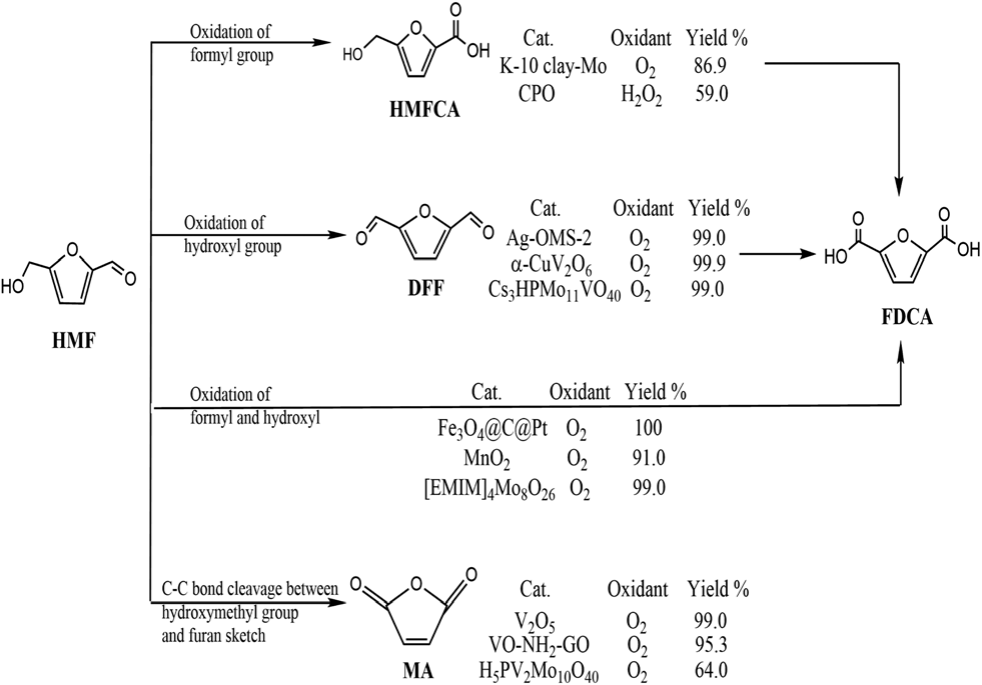
Possible competing reactions for oxidation of 5-HMF by oxygen.

With depletion of fossil resources and increasing concern about global warming, the sustainable production of fuels for transportation and useful chemicals from renewable carbon-containing biomass has attracted great attention.^1,2^ 5-Hydroxymethylfurfural (HMF) is considered a biomass-derived platform for some chemicals and has been reported to be easily dehydrated from a variety of C6-based carbohydrates.^3^ 5-HMF is considered to be biomass-derived platform for some chemicals due to the typical structure of HMF with a hydroxyl and a formyl together with a furan ring.^4^ 5-HMF could be oxidized to various products through different pathways (Scheme 1). Selective oxidation of the formyl group gives 5-hydroxymethyl-2-furancarboxylic acid (HMFCA) then further gives 2,5-furandicarboxylic acid (FDCA). Oxidation of the hydroxyl group of 5-HMF leads to the formation of 2,5-diformylfuran (DFF), which could be further oxidized to FDCA. There is a third pathway for 5-HMF oxidation: simultaneous oxidation of the formyl and hydroxyl group to give FDCA.^5^ Recently, it has been reported that 5-HMF can undergo C–C bond cleavage between the –CH_2_OH group and furan moiety and then oxidize to maleic anhydride (MA) under aerobic conditions.^6^ Therefore, oxidation of 5-HMF undergoes four pathways, leading to various products. Controlled or selective oxidation of 5-HMF is desirable for targeting production of needed products, while the selectivity to catalysts and reaction conditions including solvent, time, and temperature is essential.

Among all oxidation products, DFF is an important monomer for industrial use and has been widely used for the synthesis of various poly-Schiff bases, pharmaceuticals, macrocyclic ligands, organic conductors and polymers.^7,8^ The highest efficiency with 100% selectivity and 99% conversion was obtained with Fe_3_Co_7_/C under reaction conditions of 100 °C for 6 h.^9^ The key point of conversion of 5-HMF to DFF is to selectively oxidize the –OH group without affecting other functional groups as well as the furan ring using various catalysts and oxidants including NaOCl, BaMnO_4_, pyridinium chlorochromate, and 2,2,6,6-tetramethyl-piperidine-1-oxide.^10–12^ Based on the “green concept”, the use of oxygen or air and heterogeneous catalysts is highly favorable and desirable.^13^ Noble metals loaded on supports have been used for aerobic oxidation of 5-HMF to DFF.^14^ Also, different transition metal catalysts have been developed in 5-HMF oxidation due to their low price; however, low yield of DFF was obtained without adding any initiator.^15,16^

Polyoxometalates (POMs) are well-known redox catalysts in organic substrate transformation.^17^ Due to global energy crisis and environment pollution, it is time to decrease the consumption of fossil fuels and seek regenerated feedstocks. Therefore, biomass is regarded as a promising raw material for the sustainable production of chemicals.^18^ POMs, especially H_*n*_PMo_12−*n*_V_*n*_O_40_ (*n* = 1–6, HPA-*n*), are widely applied as oxidative catalysts in biomass conversion using H_2_O_2_ or O_2_ as oxidants, including oxidation of lignin, cellulose, and 5-HMF.^19,20^ For example, Vigier and co-workers have achieved 84% yield of 2,5-diformylfuran (DFF) from fructose in one-step oxidation by HPMo_11_V_1_O_40_.^21^ In many cases, POMs used as homogeneous catalysts faced separation problems and exhibited hydrolytic instability under oxidative conditions.^22^ The need for environmentally benign and sustainable approaches for chemical transformations has increased the focus on recyclable heterogeneous catalysts instead of homogeneous ones. Under such circumstances, heterogeneous POM catalysts are developed using microporous, mesoporous and macroporous silica materials as supports, which are used in epoxidation and oxidation of organic substrates.^23,24^ Compared to powdered mesoporous silica materials, electrospun nanofibers are good candidates because of their high surface-to-volume ratios.^25^ Therefore, highly mesostructured nanofibers are of great importance and interest for special applications in some field such as optical materials and catalyst carriers.^26,27^ SiO_2_ nanofibers are available for loading POMs using a simple sol–gel method and atom transfer radical polymerization.^28^ However, a comparatively low surface area of common electrospun silica nanofibers without a mesoporous structure is a major drawback that hinders their wide application in catalysis, where a large reaction contact area has a significant influence on the reaction rates. Therefore, it is essential to fabricate electrospun POMs/SiO_2_ nanofibers with mesoporous structure and large surface area. In this case, we have made an effort to combine electrospinning and surfactant-directed pore formation techniques to synthesize H_5_PMo_10_V_2_O_40_/SiO_2_ nanofibers with a mesoporous structure and high surface area for the first time. The benefits of H_5_PMo_10_V_2_O_40_/SiO_2_ mesoporous nanofibers over POMs/SiO_2_ mesoporous nanopowders is the high contact area, exposing more active sites for catalysis, which might result in higher reaction rates. Polyoxometalates Cs_3_HPMo_11_VO_40_ and H_3_PMo_12_O_40_/MIL-101 were used in the aerobic oxidation of 5-HMF to DFF with 99% yield at 99% conversion and 88.2% yield at 90.8% conversion, respectively, in dimethyl sulfoxide (DMSO).^29,30^ Chen *et al.* also pointed that Mo- or V-containing Keggin POMs can promote the aerobic oxidation of 5-HMF to DFF, with CsH_4_PMo_10_V_2_O_40_ and Cs_3_H_2_PMo_10_V_2_O_40_ exhibiting selectivities of 99% and 92% for DFF, respectively. Despite the higher efficiency obtained by CsH_4_PMo_10_V_2_O_40_, the problem of separation of fine powers limits its further application. Therefore, Lee's group conducted the aerobic oxidation using H_3_PMo_12_O_40_ embedded on the pore of Cr-MIL-101, giving 88.2% yield of DFF at 90.8% conversion of 5-HMF under the reaction conditions of 140 °C and 20 h,^29^ showing that there is plenty room for fabrication POM solid hybrids using other strategies for the oxidation of 5-HMF.

Oxidation of biomass by H_2_O_2_ or O_2_ has attracted much attention due to the various products available for wide application^31^ including oxidative starch,^32^ maleic anhydride from C5-biomass or C6-based polysaccharides,^33–35^ formic acid from cellulose carbohydrates,^36–40^ and 2,5-furan dicarboxylic acid (FDCA) or DFF from 5-HMF.^41,42^ The above oxidation could be achieved by using environmental benign oxidants of H_2_O_2_ or O_2_ combined with various homogeneous or heterogeneous catalysts, including metal oxides, Fe Fenton's reagents and polyoxometalates (Tables S1 and S2†). Except O_2_, H_2_O_2_ is a beneficial and environmental benign oxidant in organic transformations.^43^ In our previous work, we developed a POMs/H_2_O_2_ system in oxidation of starch.^32^ It was found that PMo_10_V_2_O_40_^5−^ was the most active species in the above reaction, but also faced separation or recycling problems. Herein, H_5_PMo_10_V_2_O_40_ was encapsulated into the pore of mesoporous silica nanofibers through surfactant-directed pore formation and electrospinning techniques. A poly(vinyl alcohol) (PVA) solution with a silica source, POMs and a surfactant were used as precursors to be electrospun and then calcined to remove the polymer and surfactant, which resulted in POMs/SiO_2_ nanofibers with a mesoporous structure. The hybrid catalysts can facilitate highly efficient oxidation of starch by H_2_O_2_ and aerobic oxidation of 5-HMF to DFF.

## 2. Experiment

### 2.1 Materials

All chemicals were used as obtained from commercial suppliers and used without further purification. EO_20_PO_20_EO_20_ (polyethylene oxide–polypropylene oxide–polyethylene oxide, P123, MW 5800) was purchased from Aldrich. 0.1 M of NaOH solution was used for determining the carboxyl content and acid strength by titration. H_5_PMo_10_V_2_O_40_ (HPMoV) was synthesized according to ref. 44 and identified by IR spectroscopy. The oxygen adsorption experiment was carried out in a closed container: 200 mg of catalyst was added in 5 mL DMSO and stirred for 0.5 h under oxygen flow. Then, the oxygen uptakes of various catalysts were investigated using a pressure gauge.

### 2.2 Catalyst characterization

IR spectra (4000–400 cm^−1^) were recorded in KBr discs on a Nicolet Magna 560 IR spectrometer. The elemental analysis was carried out using a Leeman Plasma Spec (I) ICP-ES. The ^31^P-MAS NMR measurements were obtained using a Bruker AM500 spectrometer at 202.5 MHz. Energy dispersive X-ray (EDX) analysis was performed to calculate the Si, O, P, Mo and V elements. XRD patterns of the samples were collected on a Japan Rigaku Dmax 2000 X-ray diffractometer with Cu Kα radiation (*λ* = 0.154178 nm). UV-vis spectra (200–800 nm) were recorded on a Cary 500 UV-vis-NIR spectrophotometer. DR-UV-vis spectra (200–800 nm) were obtained on a UV-2600 UV-vis spectrophotometer (Shimadzu). SEM images were determined by a SU8010 scanning electron microscope. TEM micrographs were recorded on JEM-2100F instrument. Nitrogen adsorption was measured on an ASAP 2010M surface analyzer (the samples were outgassed under vacuum at 100 °C overnight). The total organic carbon (TOC) was measured by a TOC analyzer (TOC-LCPH, Shimadzu, Japan). The O_2_ adsorption was carried out by O_2_ temperature programmed desorption. The analysis of the recovered solutions was performed using high-performance liquid chromatography (HPLC). Samples were separated by a reverse-phase C18 column and detected by a UV detector at the wavelength of 280 nm. The mobile phase constituted of acetonitrile and 0.1 wt% acetic acid aqueous solution (v/v = 10 : 90) at 0.9 mL min^−1^.

### 2.3 Preparation of HPMoV/silica precursor

The preparation of HPMoV/silica precursor was achieved as follows: P123 (1.5 g, 2.6 × 10^−4^ mol) was dissolved in ethanol (6.3 mL) at room temperature as solution A. Tetraethyl orthosilicate (TEOS, 10 g, 4.8 × 10^−2^ mol) as a structure-directed agent was added to ethanol (21.3 mL) as solution B. The desired amount of H_5_PMo_10_V_2_O_40_ was dissolved in water (2.5 mL) in the range of 0.1–0.5 g (5.8 × 10^−5^ to 2.9 × 10^−5^ mol) to ensure 5–40% H_5_PMo_10_V_2_O_40_ loading. B solution was first added to A solution, then H_5_PMo_10_V_2_O_40_ aqueous solution was added to the above mixture at room temperature under stirring. The acidity of the mixture was controlled at pH = 1–2 using HCl (12 mol L^−1^), and then the mixture was stirred vigorously for 12 h at 40 °C to give HPMoV/silica precursor.

### 2.4 Preparation of HPMoV/SiO_2_ nanofibers

10 mL of 10 wt% PVA aqueous solution was added dropwise into the above HPMoV/silica precursor, and then the mixture was heated at 60 °C for another 4 h to give the final viscous gel. The composite gel was placed in a plastic syringe. A copper pin connected to a high-voltage generator was inserted in the precursor syringe. A grounded iron drum covered with an aluminium foil served as the counter electrode. The HPMoV/SiO_2_/polymer nanofibers were prepared by electrospinning under a high voltage of 18 kV with a flow rate of 0.3 mL h^−1^. The distance of the collection plate of the aluminium foil and needle tip was 15 cm. The HPMoV/SiO_2_/polymer nanofibers were taken off from the collection plate of aluminium foil and then dried for 12 h at 60 °C under vacuum. Finally, the HPMoV/SiO_2_/polymer nanofibers were calcined at 350 °C for 8 h, giving HPMoV/SiO_2_ mesoporous nanofibers with different loading amounts of H_5_PMo_10_V_2_O_40_ (HPMoV/meso-SiO_2_(*n*-f)). Other catalysts of microporous SiO_2_ powder (micro-SiO_2_), mesoporous SiO_2_ powder (meso-SiO_2_), microporous HPMoV/SiO_2_ powder with 18% H_5_PMo_10_V_2_O_40_ loading (HPMoV/micro-SiO_2_(18)) and mesoporous HPMoV/SiO_2_ with 18% H_5_PMo_10_V_2_O_40_ loading (HPMoV/meso-SiO_2_(18)) were synthesized according to literature.^45–47^

### 2.5 Starch oxidation

In a typical reaction protocol, 1 g of starch and 4 mg of catalyst were mixed with 2.0 mL distilled water in the reactor with stirring for a period at the required temperature. The oxidation reaction was initiated by addition a certain amount of hydrogen peroxide. In order to confirm the best oxidized condtions for starch during the reaction, the total amount of hydrogen peroxide was added intermittently rather than adding the whole amount at one time. Furthermore, the amount of H_2_O_2_ added depended on the length of the experiment, usually at a rate of 900 μL per 2 h for a total reaction time of 10 h. After the reaction, the mixture was filtrated immediately to separate the insoluble catalyst and washed with water several times for reuse. The filtrate was cooled down to room temperature to separate unreacted starch by centrifugation.

### 2.6 Determination of carboxyl content^48^

300 mg of the resulting material (white powder) was dissolved in 20 mL of diluted water, and a few drops of phenolphthalein solution in EtOH were added as the indicator. The solution was titrated with 0.1 M of NaOH solution until the colour of the solution changed to pink. The degree of oxidation with respect to the percentage of carboxyl groups (mol per 100 g) presented in the polymer is calculated according to the following equation:Carboxyl content (mol per 100 g) = 100*C* × (*V* − *V*_0_)/0.3 × 1000where *C* = molar concentration of sodium peroxide, *V* − *V*_0_ = consumed volume of sodium hydroxide.

### 2.7 Aerobic oxidation of 5-HMF

Aerobic oxidation of 5-HMF was carried out in a Teflon-lined autoclave equipped with a magnetic stirrer. In a typical experiment, 1 mmol of 5-HMF and 80 mg of catalyst were added into 5 mL of DMSO; then the mixture was heated to 120 °C. During the reaction, the reactor was maintained at 1.0 MPa oxygen pressure. After the reaction, the reactor was cooled to room temperature. The solid catalyst was separated from the mixture by centrifugation and then washed with ethanol several times and dried in an oven at 40 °C for 12 h for reuse.^49^5-HMF conversion = moles of converted 5-HMF/moles of 5-HMF initially × 100%DFF yield = moles of DFF/moles of 5-HMF initial × 100%

## 3. Results and discussion

### 3.1 Preparation of POMs/SiO_2_ nanofibers with mesoporous structure

**Scheme 2:**
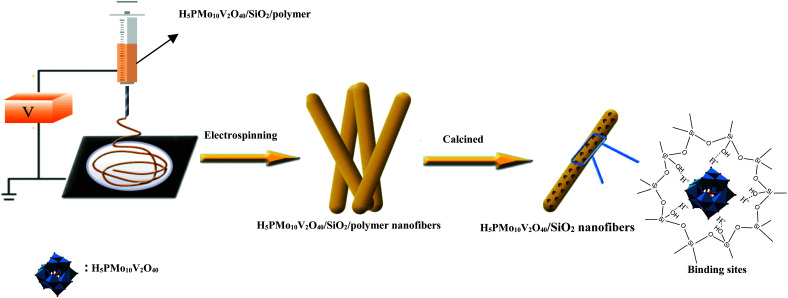
Synthesis and self-assembly of HPMoV/meso-SiO_2_(f).

Previously, Guo's group had designed mesostructured H_3_PW_12_O_40_/silica hybrids using a triblock copolymer surfactant (P123) as the pore director through sol–gel co-condensation combined with a hydrothermal method.^47^ Therefore, the preparation of H_5_PMo_10_V_2_O_40_/SiO_2_ nanofibers with a mesoporous structure used the same surfactant-directed pore formation method without hydrothermal treatment, and then *via* electrospinning to generate nanofibers (Scheme 2). First, TEOS was mixed with H_5_PMo_10_V_2_O_40_ and P123 and then hydrolyzed to form ≡(Si–OH_2_)^+^ H_4_PMo_10_V_2_O_40_ and further Po_70_EO_*m*−*y*_[(EO)·H_3_O^+^]_*y*_⋯*y*Cl^−^⋯H_5_PMo_10_V_2_O_40_/Si(OEt)_4−*n*_(OH_2_^+^)_*n*_ in the presence of strong Brønsted acid HCl.^46^ During sol formation, there had strong interaction between the POM units and the silica matrix. Second, silica sol was heated at 40 °C for 12 h, forming gel particulates. Then PVA and silica gel were spun together giving polymer/silica nanofibers. After removal of the surfactant and polymer by calcination, mesoporous H_5_PMo_10_V_2_O_40_/SiO_2_ nanofibers were formed.

### 3.2 Characterization of POMs/SiO_2_ nanofibers

**Fig. 1:**
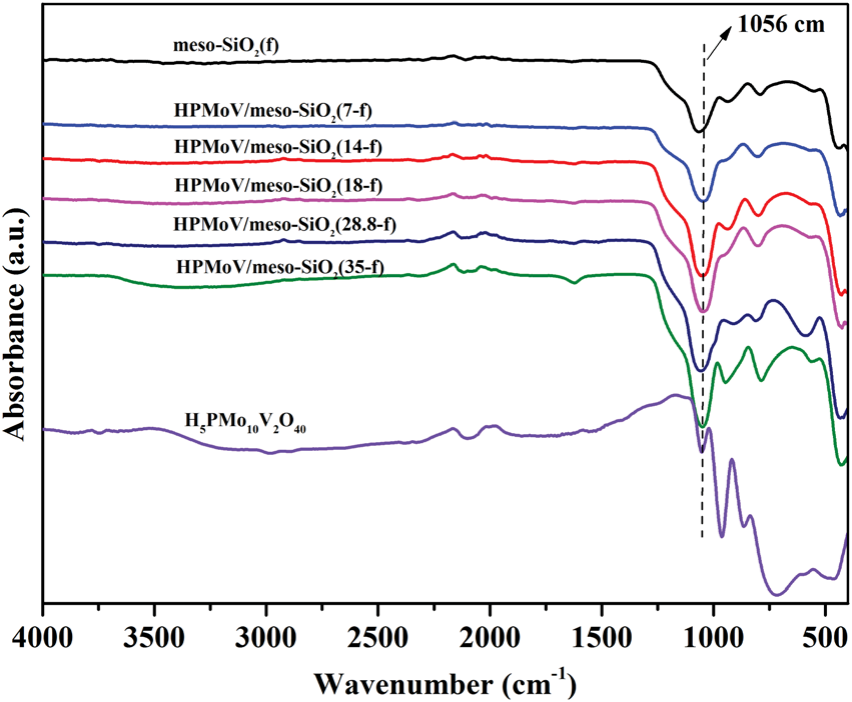
IR spectra of HPMoV/meso-SiO_2_(*n*-f).

The elemental analyses of HPMoV/meso-SiO_2_(f) with different loading amounts of H_5_PMo_10_V_2_O_40_ are given in Table 1. These results gave the molar ratio of P : Mo : V = 1 : 10 : 2, implying that H_5_PMo_10_V_2_O_40_ retained the Keggin structure during the preparation. The loading amounts of H_5_PMo_10_V_2_O_40_ in the hybrids were 7–35%. The structural integrity of the Keggin unit was further confirmed by IR spectra (Fig. 1). The IR spectra of H_5_PMo_10_V_2_O_40_ (1051, 964, 864 and 788 cm^−1^) and HPMoV/meso-SiO_2_(f) (around 1057, 956, 871 and 793 cm^−1^) gave almost the same characteristic peaks in the range of 1100–700 cm^−1^, which were assigned to the Keggin unit of *υ*_as_(P–O_a_), *υ*_as_(Mo–O_d_), *υ*_as_(Mo–O_b_–Mo), and *υ*_as_(Mo–O_c_–Mo), respectively. It could be seen that no significant interference on the IR spectrum signals originated from Keggin units, while *υ*_as_(P–O_a_), *υ*_as_(Mo–O_d_), *υ*_as_(Mo–O_b_–Mo), and *υ*_as_(Mo–O_c_–Mo) were observed with some blue shifts after the formation of HPMoV/SiO_2_ materials. Such shifts of the IR peaks were due to the strong interactions between POM anion and silica support. Meanwhile, the stretching vibration of Si–O–Si for the SiO_2_ support was observed at 1084 cm^−1^,^50^ and a broad peak at 1073 cm^−1^ was found in HPMoV/meso-SiO_2_(f) due to the strong interaction of the Keggin anion and SiO_2_ support.

**Table 1:** Elementary analysis, loading of H_5_PMo_10_V_2_O_40_, BET surface areas, and porosity data for H_5_PMo_10_V_2_O_40_ and HPMoV/SiO_2_ hybrids

Catalyst	Elementary results (calculated values in parenthesis)/%				Loading amount%	*S*_BET_ (m^2^ g^−1^)	*V*_p_ a (cm^3^ g^−1^)	*D*_p_ b (nm)
	P	Mo	Si	V				
H_5_PMo_10_V_2_O_40_	1.8(1.78)	55(55.2)	—	5.8(5.86)	100	18.0	0.11	0.72
HPMoV/micro-SiO_2_	1.49(1.50)	46.2(46.32)	7.51(7.53)	5.0(4.94)	18	445.9	0.35	3.41
Meso-SiO_2_	—	—	46.73(46.74)	—	—	736.2	0.21	5.31
HPMoV/meso-SiO_2_	1.48(1.50)	46.3(46.32)	7.52(7.53)	4.9(4.90)	18	431.6	0.32	3.34
SiO_2_(f)	—	—	46.71(46.74)	—	—	322.7	0.33	3.32
meso-SiO_2_(f)	—	—	46.72(46.74)	—	—	433.5	0.38	3.92
HPMoV/meso-SiO_2_(7-f)	1.20(1.19)	37.0(36.96)	15.5(15.46)	3.9(3.92)	7	428.8	0.37	3.86
HPMoV/meso-SiO_2_(14-f)	1.42(1.43)	44.3(44.3)	9.3(9.26)	4.5(4.70)	14	407.2	0.39	3.72
HPMoV/meso-SiO_2_(18-f)	1.48(1.50)	46.3(46.32)	7.5(7.53)	5.0(4.91)	18	333.3	0.35	3.86
HPMoV/meso-SiO_2_(28.8-f)	1.60(1.59)	49.4(49.3)	5.0(5.0)	5.25(5.24)	28.8	268.5	0.30	3.98
HPMoV/meso-SiO_2_(35-f)	1.65(1.62)	50.3(50.26)	4.0(4.12)	5.4(5.34)	35	255.5	0.28	3.16
HPMoV/meso-SiO_2_(18-f) after reaction	1.41(1.45)	45.3(46.32)	8.7(7.53)	4.5(4.9)	18	362.5	0.37	3.60

a Pore volume (*V*_p_) was estimated from the pore volume determination using the adsorption branch of the N_2_ isotherm curve at *P*/*P*_0_ = 0.99 single point.

b Pore diameter (*D*_p_) was estimated from BJH adsorption determination.

**Fig. 2:**
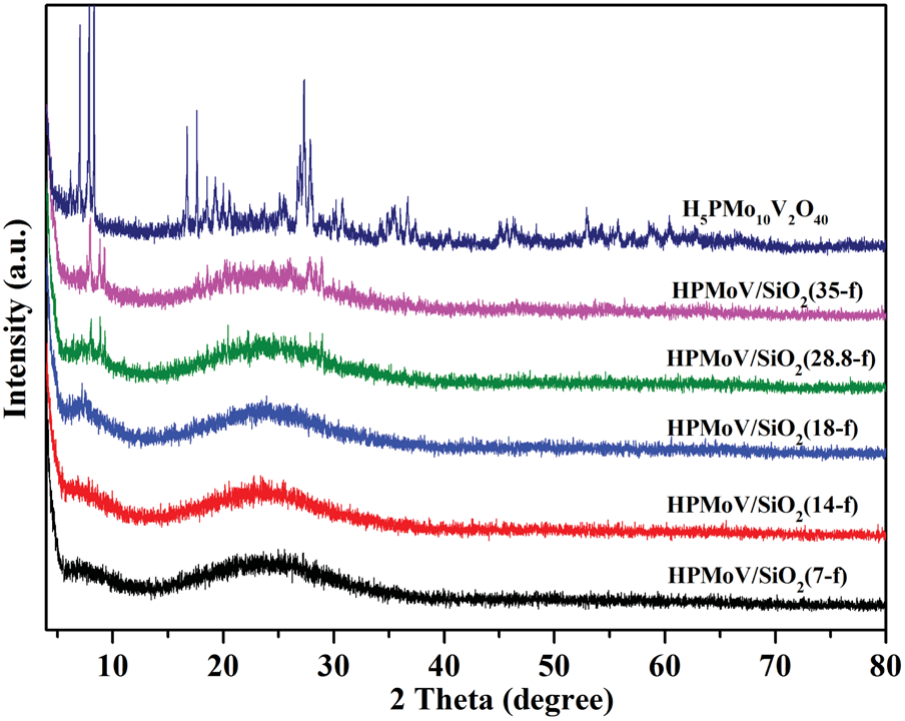
Wide-angle XRD patterns of HPMoV/meso-SiO_2_(*n*-f).

The wide-angle XRD patterns of HPMoV/meso-SiO_2_(f) were showed in Fig. 2, which were used to study the dispersion of the Keggin POM unit throughout the composites (Fig. 2). A broad diffraction peak at 2*θ* of 15–35° was observed for the nanofibers with H_5_PMo_10_V_2_O_40_ loading lower than 18 wt%, which was assigned to amorphous silica. There were no diffraction peaks corresponding to the starting H_5_PMo_10_V_2_O_40_ in HPMoV/meso-SiO_2_(*n*-f) nanofibers with H_5_PMo_10_V_2_O_40_ loading lower than 18 wt%. When the loading amount was increased to 28.8 wt% or more, diffraction peaks for H_5_PMo_10_V_2_O_40_ were found at 8.86° (101), 9.24° (002), 18.62° (212), 28.01° (323). The above results indicated that H_5_PMo_10_V_2_O_40_ molecules were homogeneously dispersed in the composites with lower loading amounts (<18 wt%), while they were unevenly dispersed or aggregated across the nanofibers at higher loading amounts. The DR-UV-vis spectra of the HPMoV/meso-SiO_2_(*n*-f) are given in Fig. S1.† The characteristic peak of the Keggin anion at 215 nm was found for all HPMoV/meso-SiO_2_(*n*-f), which was attributed to the electron transmission of molybdenum to oxygen. This proved that H_5_PMo_10_V_2_O_40_ was successfully modified on the mesoporous SiO_2_ nanofibers.

**Fig. 3:**
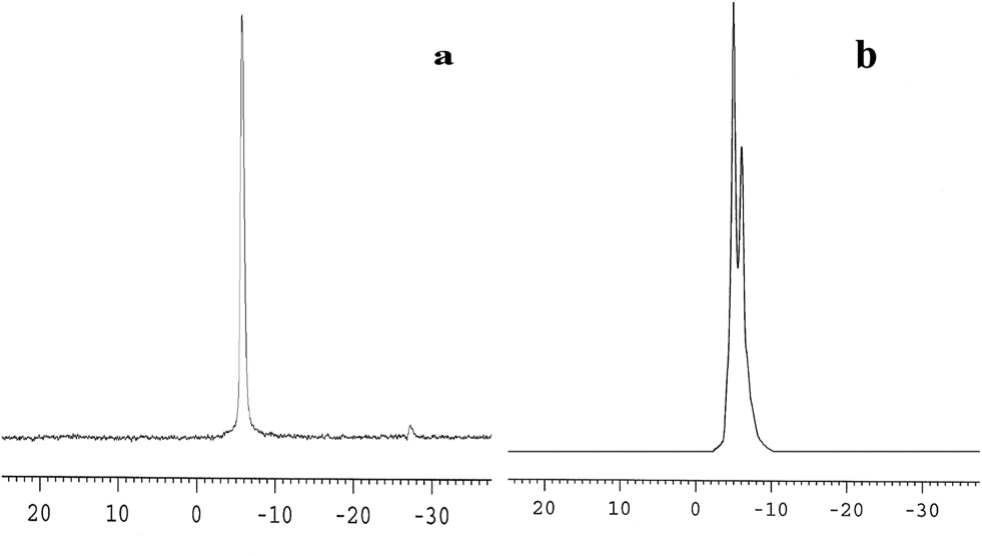
^31^P-MAS NMR spectra of H_5_PMo_10_V_2_O_40_ (a) and HPMoV/meso-SiO_2_(18-f) (b).

**Fig. 4:**
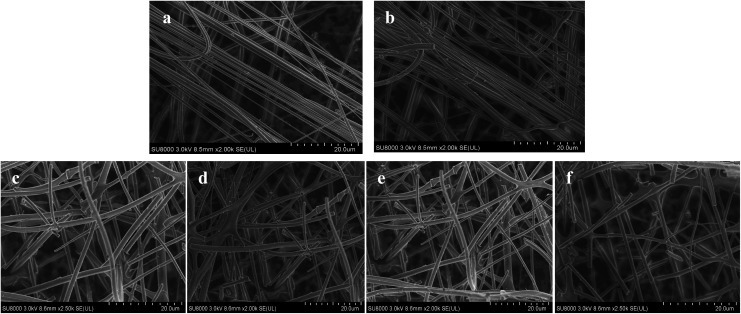
SEM images of HPMoV/SiO_2_/polymer (a), HPMoV/meso-SiO_2_(7-f) (b), HPMoV/meso-SiO_2_(14-f) (c), HPMoV/meso-SiO_2_(18-f) (d), HPMoV/meso-SiO_2_(28.8-f) (e), and HPMoV/meso-SiO_2_(35-f) (f).

**Fig. 5:**
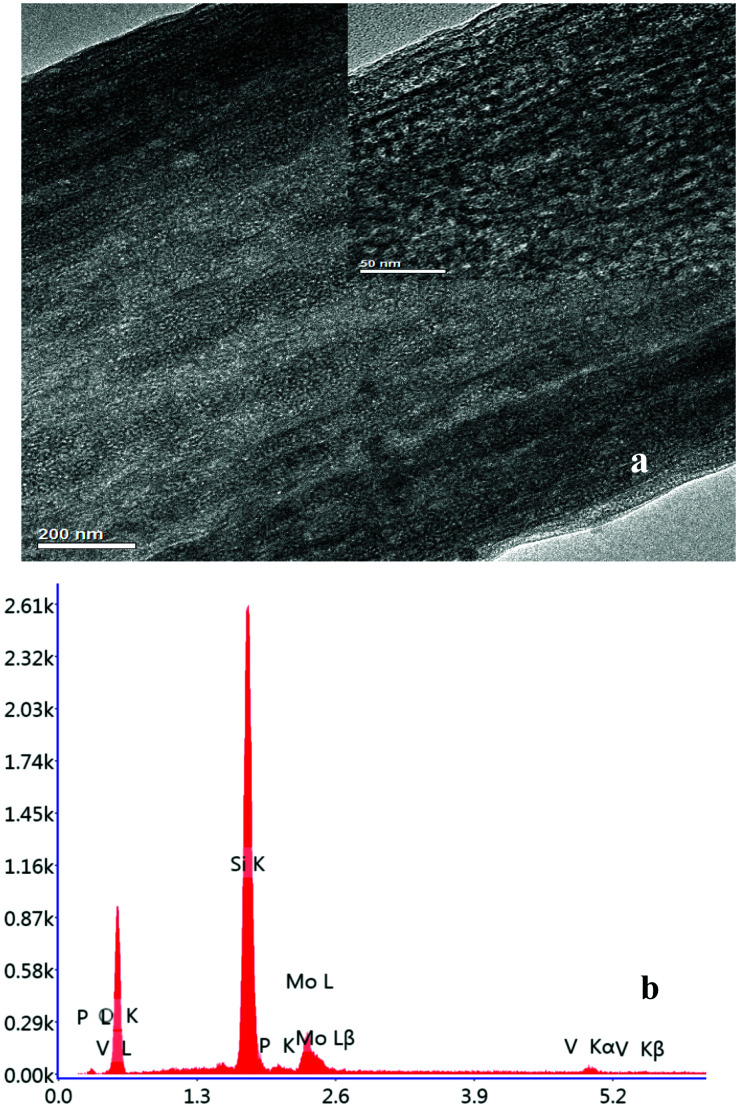
TEM image of HPMoV/meso-SiO_2_(18-f) nanofibers (a) and EDX pattern (b).

The ^31^P-MAS NMR was used to confirm the structural integrity of the Keggin anion in silica nanofibers and the presence of the interaction between silica and POMs (Fig. 3). The ^31^P-NMR of HPMoV/meso-SiO_2_(18-f) gave two peaks at −4.88 and −5.96 ppm (Fig. 3b). A peak at −4.88 ppm was attributed to the existence of PMo_10_V_2_O_40_^5−^ (−4.55 ppm),^51^ indicating the structural integrity of the Keggin unit in silica nanofibers. A small peak at −5.96 ppm confirmed the strong interaction of Si–O–W occurrence, which permitted less leaching of POMs from the support. The SEM images of HPMoV/SiO_2_/polymer and HPMoV/meso-SiO_2_(*n*-f) are given in Fig. 4. It can be seen that the HPMoV/SiO_2_/polymer consisted of uniform fibers with a diameter of 400–500 nm and good dispersity. After calcination at 350 °C for HPMoV/SiO_2_/polymer to remove the polymer and surfactant, the as-prepared HPMoV/SiO_2_ hybrids shrank to 200–400 nm in diameter due to the decomposition of PVA and gelation of TEOS. In addition, the TEM images (Fig. 5) of HPMoV/meso-SiO_2_(18-f) revealed that HPMoV/meso-SiO_2_(18-f) was three-dimensionally interconnected pore-network structure with poor ordering but high porosity.^47^ Meanwhile, H_5_PMo_10_V_2_O_40_ was observed as dark spots on SiO_2_ with uniform dispersion.

**Fig. 6:**
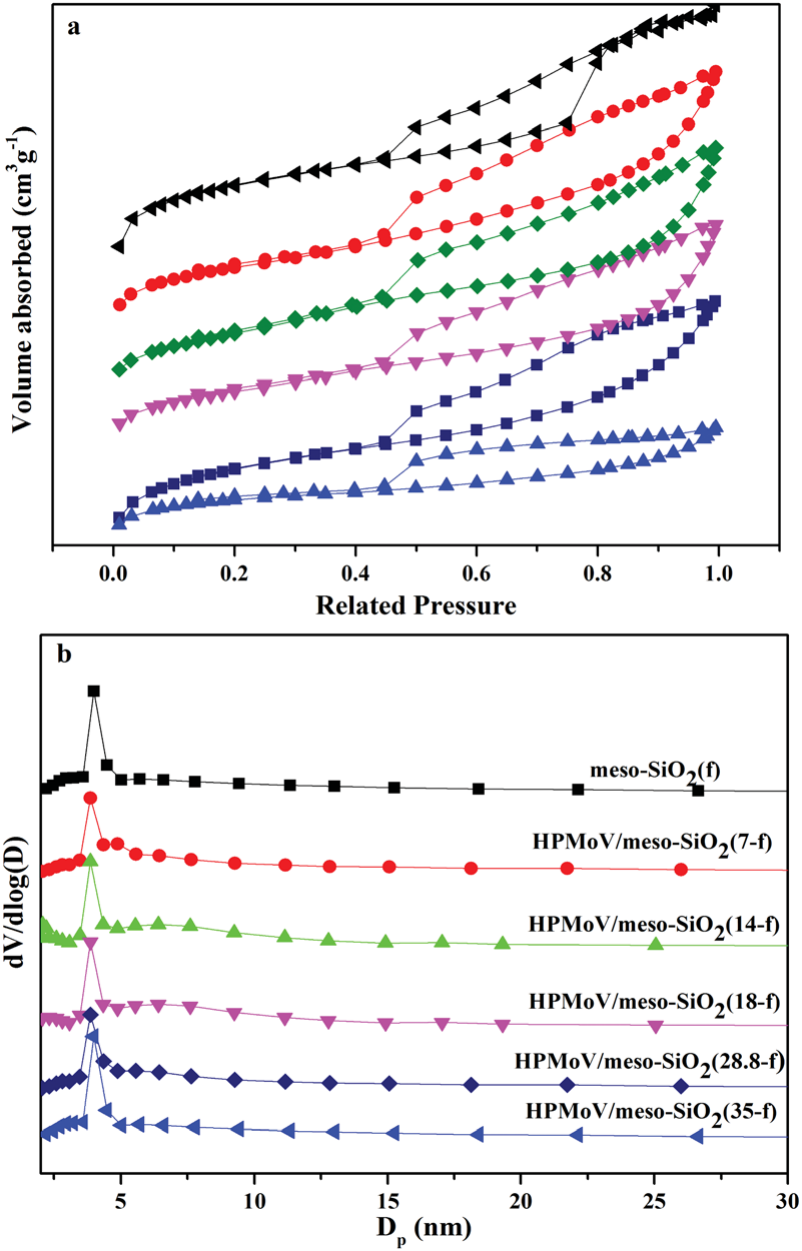
Nitrogen adsorption–desorption isotherms (a) and pore size distribution profiles according to BJH desorption d*V*/d*D* pore volume (b) of HPMoV/meso-SiO_2_(*n*-f).

The pore sizes and N_2_ porosimetry results of HPMoV/meso-SiO_2_(*n*-f) are shown in Fig. 6. The isotherm of all hybrid catalysts can be classified as type IV, which is characteristic of a mesoporous structure (Fig. 6a). It found that the catalysts exhibited H_1_ hysteresis loops at a relative pressure (*P*/*P*_0_) of 0.45–0.90, indicating the hybrid catalysts had regular mesopores.^52^ This result was consistent with the XRD and TEM results and further proved the mesostructure of HPMoV/meso-SiO_2_(*n*-f). From Fig. 6b, the pore size distributions of the HPMoV/meso-SiO_2_(*n*-f) materials had uniform pore sizes and the Keggin units were homogeneously distributed across the sol–gel co-condensed materials.

### 3.3 Oxidation of starch by H_2_O_2_

**Fig. 7:**
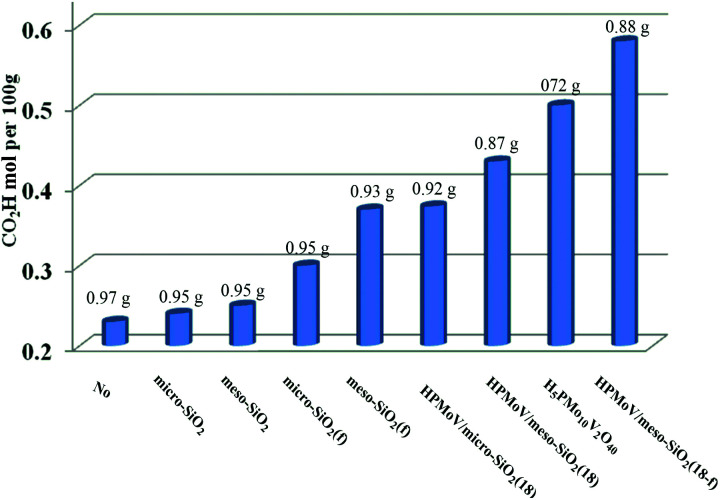
Influence of catalyst structure on the catalytic performance in starch oxidation. Reaction conditions: starch (1 g), catalyst (4 mg), H_2_O (2 mL), H_2_O_2_ (4 mmol), temperature (70 °C), reaction time (10 h).

First, HPMoV/meso-SiO_2_(*n*-f) were used in oxidation of starch by H_2_O_2_ under the reaction conditions: 1 g of starch, 2 mL of distilled water, 4.5 mL of hydrogen peroxide (30%), and 4 mg of catalyst at 70 °C for 10 h. In order to evaluate the influence of structure, pore size and morphology on catalytic activity, various catalysts were selected to be compared including micro-SiO_2_, meso-SiO_2_, micro-SiO_2_(f), meso-SiO_2_(f), HPMoV/micro-SiO_2_(18), HPMoV/micro-SiO_2_(18-f), HPMoV/meso-SiO_2_(18), HPMoV/meso-SiO_2_(18-f) and H_5_PMo_10_V_2_O_40_. It can be seen that without any catalysts (Fig. 7), starch could not be oxidized by H_2_O_2_ to a large extent and only gave carboxyl content of 0.23 mol per 100 g. Adding SiO_2_ did not enhance the carboxyl content significantly, showing that silica materials were not actual active sites for oxidation reactions. The oxidation degree of starch was increased using HPMoV/SiO_2_ hybrids with a micro- or meso-porous structure, showing that H_5_PMo_10_V_2_O_40_ was the active site for the oxidation reaction. From Fig. 7, it could also be seen that HPMoV/SiO_2_ with a mesoporous structure gave higher activity than HPMoV/SiO_2_ with a microporous structure, which was due to the large size of the starch molecules. Interestingly, it was found that HPMoV/meso-SiO_2_ materials with nanofiber morphology exhibited higher oxidation activity than those without one-dimensional morphology, which was because the nanofiber structure could adsorb more substrates than others with different morphologies. The essential adsorption of starch by HPMoV/meso-SiO_2_(f) was determined by IR spectrum of HPMoV/meso-SiO_2_(18-f) after absorbing starch (Fig. S2c†). It could be seen that two characteristic peaks of starch at 2921 cm^−1^ and 1153 cm^−1^ were observed, while that at 1167 cm^−1^ for the original glucose unit shifted to 1153 cm^−1^. Meanwhile, the peaks belonging to the terminal O attached to metals of Keggin POMs changed from 1052 to 1044 cm^−1^. All these results indicated the occurrence of interaction between POMs and starch molecules, which the main contributor to enhancement in activity of solid POM catalysts. HPMoV/meso-SiO_2_(18) without nanofiber morphology could also adsorb some starch, but the amount of adsorbed starch was different, as 0.3 g g^−1^ for HPMoV/meso-SiO_2_(18) compared to 0.5 g g^−1^ for HPMoV/meso-SiO_2_(18-f), due to the nanofiber structure exhibiting sufficient length, high porosity and ultrahigh surface-to-volume ratio could adsorb more starch.^53^ HPMoV/meso-SiO_2_(18) without nanofiber morphology presented a BET surface area of 431.6 m^2^ g^−1^ (Table 1), which was higher than that of HPMoV/meso-SiO_2_(18-f). Therefore, the difference in catalytic activity between the nanofibers and nanoparticles of HPMoV/SiO_2_ was expected to have different morphologies. At last, HPMoV/meso-SiO_2_(18-f) presented the highest oxidation activity with a carboxyl content as 0.58 mol per 100 g among all catalysts, including the homogeneous one H_5_PMo_10_V_2_O_40_, silica materials, and other HPMoV/silica catalysts without a nanofiber morphology. Meanwhile, the solid products were obtained with different yields; homogenous H_5_PMo_10_V_2_O_40_ only gave 72.0% yield of solid oxidative starch and HPMoV/meso-SiO_2_(18-f) presented 88.0% yield. This indicated that after being loaded on meso-SiO_2_, some active sites become embedded, which could hinder further oxidation occurrence.

The difference between homogeneous H_5_PMo_10_V_2_O_40_ and HPMoV/meso-SiO_2_(*n*-f) was attributed to the different decomposition rates for H_2_O_2_ (Fig. S3a†); H_2_O_2_ was decomposed faster by H_5_PMo_10_V_2_O_40_ than by HPMoV/meso-SiO_2_(*n*-f). This result indicated that active sites of H_5_PMo_10_V_2_O_40_ were surrounded by the Si–O matrix, resulting in lower decomposition rate for H_2_O_2_, while the decomposition of H_2_O_2_ depended on its loading amount on silica. In our previous report,^47,48^ the oxidation of starch underwent singlet oxygen and OH˙ radical mechanism, while the decomposition of H_2_O_2_ to O_2_ could hinder the oxidation of starch. Therefore, two competing reactions based on H_2_O_2_ including oxidation of starch and decomposition into O_2_ were balanced by HPMoV/meso-SiO_2_(18-f). The H_5_PMo_10_V_2_O_40_ molecule was embedded in to silica, which could prevent the decomposition of H_2_O_2_ and HPMoV/meso-SiO_2_(18-f) gave the highest carboxyl content of 0.58 mol per 100 g. HPMoV/meso-SiO_2_(18) without nanofibers showed higher activity in H_2_O_2_ decomposition than nanofibers, which was due to its higher BET surface area than nanofibers. The utilization of H_2_O_2_ using H_5_PMo_10_V_2_O_40_, HPMoV/meso-SiO_2_(7-f), HPMoV/meso-SiO_2_(14-f), HPMoV/meso-SiO_2_(18-f), HPMoV/meso-SiO_2_(28.8-f), and HPMoV/meso-SiO_2_(35-f) could confirm this point (Fig. S3b†). The utilization of H_2_O_2_ for HPMoV/meso-SiO_2_ depended on the loading amount of H_5_PMo_10_V_2_O_40_ on meso-silicas in order of HPMoV/meso-SiO_2_(18-f) > HPMoV/meso-SiO_2_(18) > HPMoV/meso-SiO_2_(14-f) > HPMoV/meso-SiO_2_(7-f) > HPMoV/meso-SiO_2_(28.8-f) > HPMoV/meso-SiO_2_(35-f) > H_5_PMo_10_V_2_O_40_, while the utilization of H_2_O_2_ was measured by the titration of Ce(SO_4_)_2_ in oxidation of starch.^54^

**Fig. 8:**
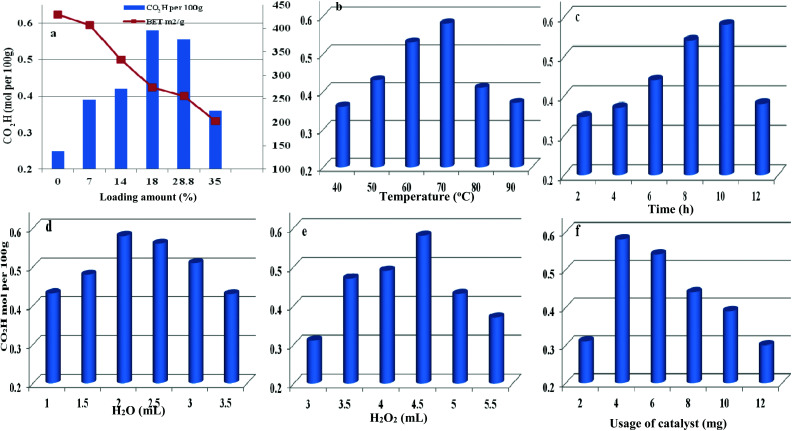
Parameters affecting the oxidation reaction including different H_5_PMo_10_V_2_O_40_ loading (a), temperature (b), time (c), water content (d), H_2_O_2_ dosage (e), and usage of catalyst (f).

The loading amount of H_5_PMo_10_V_2_O_40_ influenced the oxidative degree of starch in Fig. 8a. By increasing the loading amount of H_5_PMo_10_V_2_O_40_ from 0 to 18 wt%, the carboxyl content was increased from 0.25 to 0.58 mol per 100 g, respectively. However, the carboxyl content was not enhanced on further increasing the H_5_PMo_10_V_2_O_40_ loading. The decreasing trend was attributed to the decrease in BET surface area and pore sizes with increase in H_5_PMo_10_V_2_O_40_ loading and the decomposition of H_2_O_2_ had a negative effect on starch oxidation.

Therefore, HPMoV/meso-SiO_2_(18-f) were used as catalysts in the oxidation of starch and other reaction conditions were optimized (Fig. 8b–f). Other parameters including water content, usage of HPMoV/meso-SiO_2_(18-f), temperature and H_2_O_2_ dosage were also studied in Fig. 8. From Fig. 8b, it could be seen that oxidation of starch depended on the changes in temperature and the carboxyl content increased with increasing temperature from 40 to 70 °C. Then, further increasing reaction temperature led to a decrease in oxidative degree, which was due to the decomposition of H_2_O_2_ and starch gelatinization at high temperature.^55^Fig. 8c shows the effect of reaction time on the carboxyl content of the starch, which reached the maximum value of 0.58 mol per 100 g at 10 h. Fig. 8d shows the influence of water usage on the activity. It can be seen that the carboxyl content of oxidized starch increased with water content from 1.0 mL to 2.0 mL, which was attributed to the decrease in viscosity of the starch solution. Generally, the diameter of the starch molecule was approximately 2–5 μm. With increasing in water content from 1.0 mL to 2.0 mL, the apparent viscosity of starch decreased and fluidity increased, leading to easier access to active sites in the nanofibers. The SEM images of starch with different water contents (Fig. S4†) verified the above results. However, the oxidation performance was not desirable by further increase of water content because excess water can decrease the concentration of H_2_O_2_. The usage of H_2_O_2_ was also optimized (Fig. 8e), while the carboxyl content increased significantly from 0.31 to 0.58 mol per 100 g, corresponding to 3.0 mL and 4.5 mL of H_2_O_2_, respectively. Further increasing to 5 mL or more volume of H_2_O_2_ did not give increasing of oxidative degree, which was attributed to that the decomposition of H_2_O_2_ to O_2_ at 70 °C might be accelerated when using high amount of H_2_O_2_ (Fig. S5†). It could be seen that the decomposition rates of H_2_O_2_ at usages of 5.0 and 5.5 mL were higher those at other usages. The utilization of H_2_O_2_ in oxidation of starch varied according to the usage of H_2_O_2_ under HPMoV/meso-SiO_2_(18-f) (Fig. S6†). Therefore, further increasing of H_2_O_2_ usage did not lead to an increasing in the carboxyl content. The influence of usage of HPMoV/meso-SiO_2_(18-f) on activity is given in Fig. 8f. It could be seen that increasing the usage of the catalyst accelerated the oxidation degree and then further increasing more amount of catalyst slowed down the oxidation degree. Therefore, the maximum value was obtained at 4 mg of HPMoV/meso-SiO_2_(18-f). This result further demonstrated that there were two competing reactions in oxidation by H_2_O_2_: one is the oxidation of starch and the other is the decomposition to H_2_O_2_ to O_2_ and H_2_O. It could be concluded that increasing the usage of catalyst resulted in faster H_2_O_2_ decomposition and 4.0 mg of HPMoV/meso-SiO_2_(18-f) was the best choice. Finally, the optimized conditions for oxidation of starch through HPMoV/meso-SiO_2_(18-f) and H_2_O_2_ were 1 g of starch, 2 mL of distilled water, 4.5 mL of hydrogen peroxide (30%), and 4 mg of catalyst at 70 °C for 10 h, obtaining a carboxyl content as high as 0.58 mol per 100 g. In comparison with the reported POMs and FeSO_4_ (Table S2†), it could be concluded that HPMoV/SiO_2_ nanofibers with a mesoporous structure exhibited higher oxidation activity of starch and lower decomposition of H_2_O_2_.

### 3.4 Oxidation of 5-HMF through POM hybrids and oxygen

**Fig. 9:**
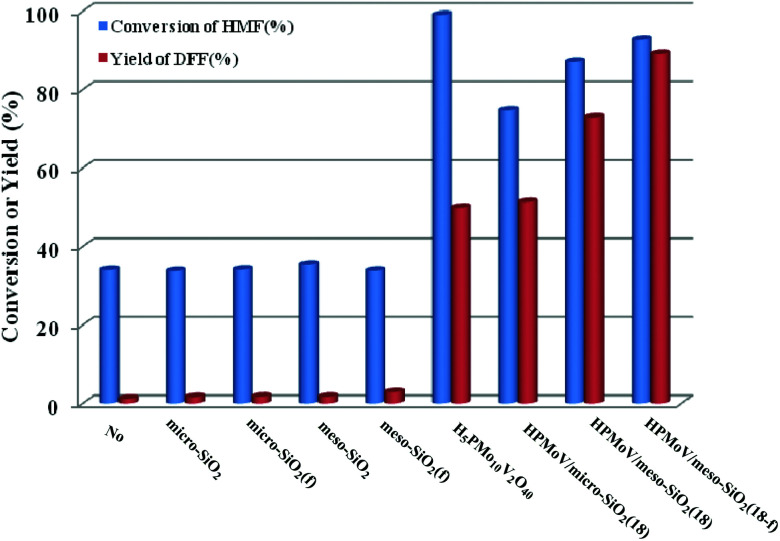
Catalytic performance for various catalysts with different structures in oxidation of 5-HMF by O_2_. Reaction conditions: 1 mmol of 5-HMF, 80 mg of catalyst, and 5 mL solvent at 120 °C for 8 h.

**Fig. 10:**
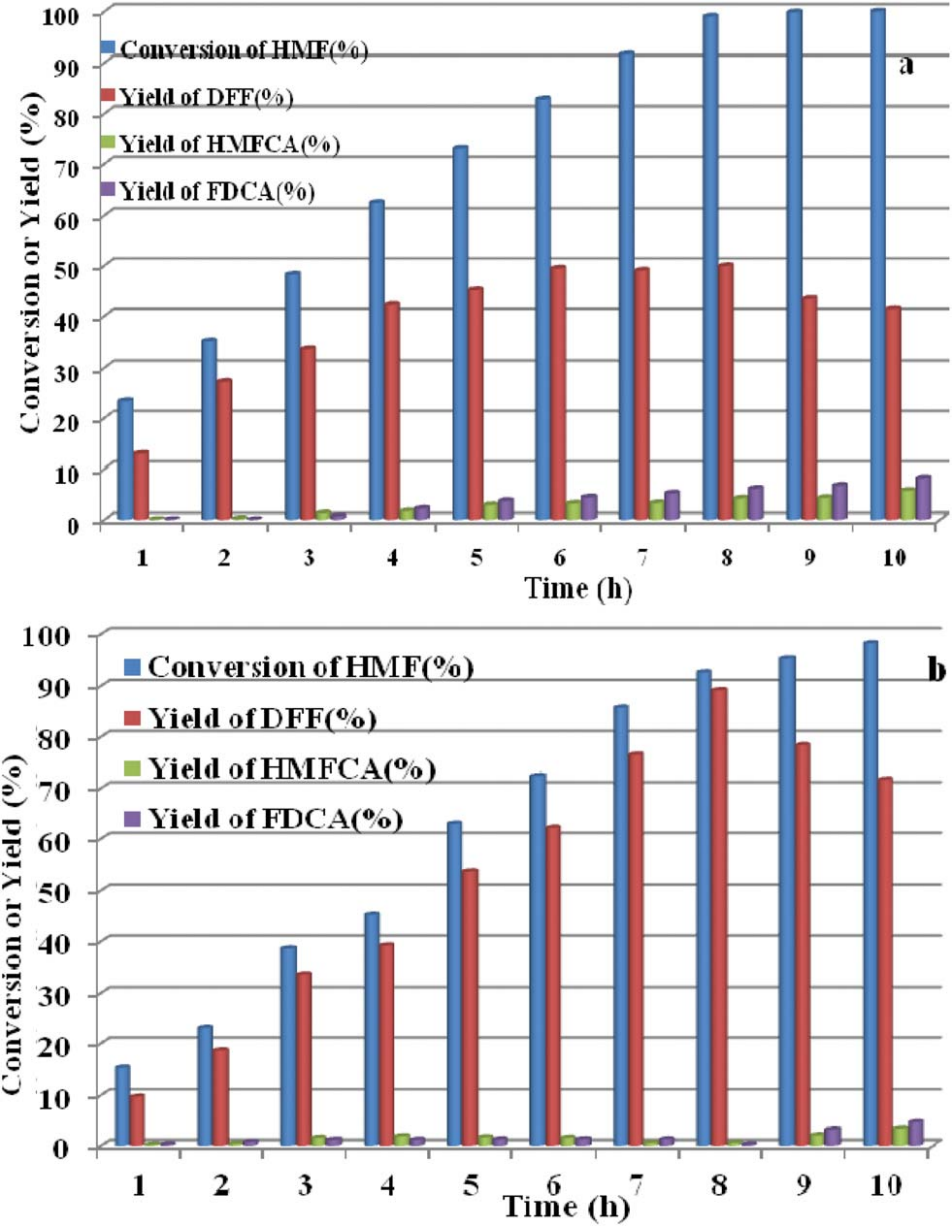
Conversion of 5-HMF and product distribution for H_5_PMo_10_V_2_O_40_ (a) and HPMoV/meso-SiO_2_(18-f), (b) verse reaction time under reaction conditions of: 1 mmol of 5-HMF, 80 mg of catalyst, and 5 mL solvent at 120 °C for 8 h.

HPMoV/meso-SiO_2_(*n*-f) were successfully used as catalysts in H_2_O_2_ oxidation of starch. Oxygen is regarded as a more environmental benign oxidant than H_2_O_2_ in organic transformation. Therefore, catalyzing O_2_ to oxidize bio-platform chemicals is of great value. The different activity on 5-HMF oxidation by O_2_ was achieved using various solid catalysts including micro-SiO_2_, meso-SiO_2_, micro-SiO_2_(f), meso-SiO_2_(f), HPMoV/micro-SiO_2_(18), HPMoV/micro-SiO_2_(18-f), HPMoV/meso-SiO_2_(18), HPMoV/meso-SiO_2_(18-f) and H_5_PMo_10_V_2_O_40_ under the reaction conditions of 1 mmol of 5-HMF and 80 mg of catalyst at 120 °C for 8 h (Fig. 9). It could be seen that 5-HMF could be oxidized without any catalyst or with silica materials as catalysts, but little DFF was found in all cases. Because of no differential between no catalyst and silica, it could be concluded that silica material showed no catalytic performance toward 5-HMF oxidation by O_2_. The reaction process was accelerated when H_5_PMo_10_V_2_O_40_ or HPMoV/SiO_2_ hybrids were used as catalysts. H_5_PMo_10_V_2_O_40_ is a homogeneous catalyst, giving 99% conversion of 5-HMF but 50% selectivity to DFF. The higher conversion of 5-HMF by H_5_PMo_10_V_2_O_40_ was attributed to its higher redox potential, which is favorable for 5-HMF oxidation, whereas its stronger redox ability gave rise to lower selectivity to DFF. Checking the product distribution verse reaction time in presence of H_5_PMo_10_V_2_O_40_ (Fig. 10a), it was found that the oxidation of 5-HMF underwent a series of competing reactions including (1) 5-HMF → HMFCA → FDCA; (2) 5-HMF → DFF → FDCA; and (3) 5-HMF → CO_2_ with a high extent of oxidation. Prolonging the reaction time led to almost 100% 5-HMF conversion and generated a high amount of HMFCA, FDCA and CO_2_, while a yield as high as 49.9% was obtained for DFF at reaction time of 8 h. The deep oxidation occurred being measured by TOC measurement (Fig. S7†), which prolonged the reaction time, leading to a higher loss of TOC by H_5_PMo_10_V_2_O_40_. This indicated strong redox ability of homogeneous H_5_PMo_10_V_2_O_40_, leading to over-oxidation of DFF to CO_2_. In order to obtain DFF with a higher yield, solid or loaded POMs are needed. From Fig. 9, it can be seen that HPMoV/micro-SiO_2_(18) and HPMoV/meso-SiO_2_(18) nanopowders were more active than pure silica supports, while the DFF yields were 33.9% and 2.9% at 51.3% and 72.9% conversions, respectively, under the same reaction conditions 1 mmol of 5-HMF and 80 mg of catalyst at 120 °C for 8 h). Using HPMoV/meso-SiO_2_(18-f), significant improvement in DFF yield to 89.2% at 92.7% conversion was achieved. Due to H_5_PMo_10_V_2_O_40_ embedded on the silica matrix, the over-oxidation of DFF to FDCA even CO_2_ was effectively prevented (Fig. 10b). Increasing reaction time to 9 or 10 h only resulted in 10.8% and 16.4% decrease in yields of DFF, respectively, while generation of CO_2_ was also limited compared to that obtained using homogeneous H_5_PMo_10_V_2_O_40_. Therefore, it could be said that the silica support played a positive role in DFF production as selectivity to DFF by HPMoV/SiO_2_ was higher than by pure H_5_PMo_10_V_2_O_40_. Compared to the two reports concerning POMs including Cs_3_HPMo_11_V (TOF = 241.8 mol mol^−1^ h^−1^, TOF was calculated as (amount of DFF, mol)/[(amount of polyoxometalates, mol) × (reaction time, h)] at 120 °C and H_3_PMo_12_O_40_ (PMA)-MIL-101 (TOF = 163.2 mol mol^−1^ h^−1^) at 140 °C, and an enhanced TOF value of 242.1 mol mol^−1^ h^−1^ at 120 °C was obtained for HPMoV/meso-SiO_2_ nanofibers.

In comparison with microporous and mesoporous HPMoV/SiO_2_(18) nanopowders, HPMoV/meso-SiO_2_(18) nanofibers presented improved activity with almost 37.9% and 16.2% increase in DFF yield, respectively. This improvement was attributed to the effect of nanofiber morphology of solid catalysts on the uptake amount of oxygen. The oxygen uptake of different catalysts is shown in Table S3.† From Table S3,† the uptake amounts of oxygen significantly increased after H_5_PMo_10_V_2_O_40_ (5.06 × 10^−7^ mol g^−1^) loading on the SiO_2_ support. HPMoV/meso-SiO_2_(18) nanofiber and HPMoV/meso-SiO_2_(18) nanopowder content was 5.6 × 10^−5^ mol g^−1^ and 4.3 × 10^−5^ mol g^−1^, respectively, which showed almost 1.3 times enhancement. Meso-SiO_2_ nanofibers with a hierarchical mesoporous internal structure possessed gas-adsorption characteristics.^56^ As shown in Fig. S8,† the desorption peak at 100–200 °C could be ascribed to O species loosely bound to the surface of the catalyst.^57^ From Fig. S8,† HPMoV/meso-SiO_2_ nanofibers were prone to uptake oxygen, which provided more accessibility for 5-HMF being oxidized by O_2_.

**Fig. 11:**
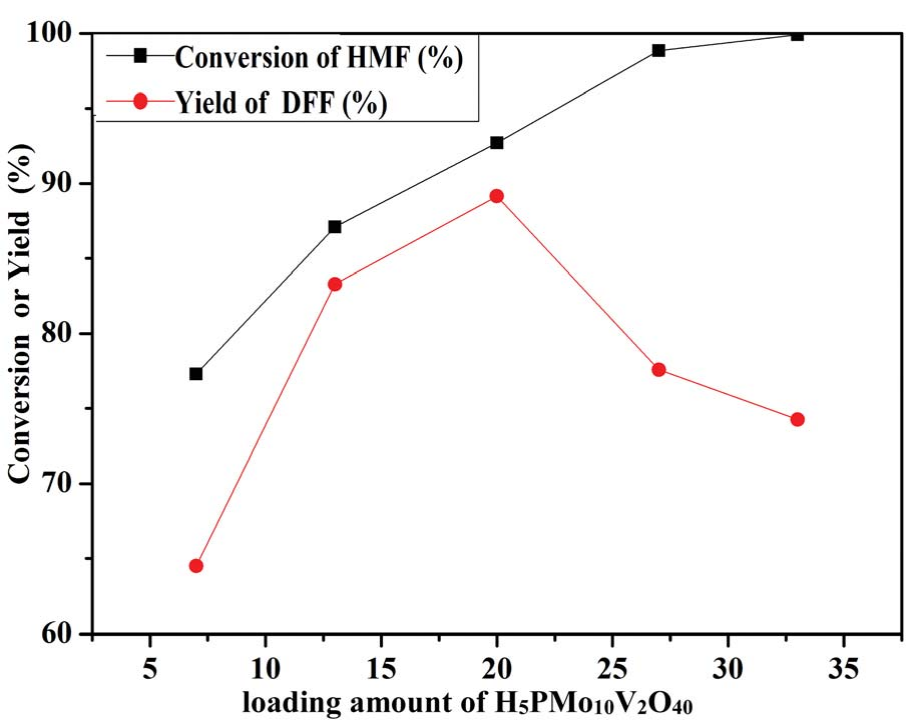
Different loading amounts of H_5_PMo_10_V_2_O_40_ on mesoporous SiO_2_ nanofibers on oxidation of 5-HMF under the reaction conditions of 1 mmol of 5-HMF, 80 mg of catalyst, and 5 mL solvent at 120 °C for 8 h.

The oxidation of 5-HMF depended on the loading amount of H_5_PMo_10_V_2_O_40_ on SiO_2_ mesoporous nanofibers (Fig. 11). 77.3% 5-HMF conversion and 64.5% DFF yield were obtained when HPMoV/meso-SiO_2_(7-f) was used as the catalyst. A maximum yield of 89.2% at 92.7% conversion was obtained using HPMoV/meso-SiO_2_(18-f). Then on further increasing the loading of H_5_PMo_10_V_2_O_40_, the yield of DFF decreased to 77.6% only with slight increase in 5-HMF conversion. This result showed that higher H_5_PMo_10_V_2_O_40_ loading could promote the conversion of 5-HMF, but also led to a decrease in the BET surface area and pore size, which were unfavorable for the interaction between the reactant and active sites. Meanwhile, higher loading of H_5_PMo_10_V_2_O_40_ led to lower selectivity to DFF, which was due to the over-oxidation of DFF to FDCA.

**Fig. 12:**
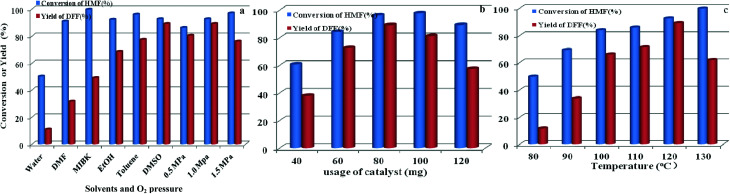
Parameters in the oxidation of HFM including solvents and O_2_ pressure (a), usage of catalyst (b), and reaction temperature (c) using 1 mmol of 5-HMF at 120 °C for 8 h.

The effects of other parameters including solvent and O_2_ pressure, usage of catalyst, and reaction temperature on catalytic activity by O_2_ and HPMoV/meso-SiO_2_(18-f) were also studied. As shown in Fig. 12a, the influence of solvents on aerobic oxidation of 5-HMF was checked using various solvents including water, DMSO, toluene, ethanol, methyl isobutyl ketone (MIBK), and *N*,*N*-dimethylformamide (DMF). In the water system, the catalytic activity was negligible with 11.0% yield of DFF at 50.1% conversion of 5-HMF, presumably due to the hydration of the aldehyde group in 5-HMF to geminal diols in water.^58^ In organic solvents, the conversion of HMF followed the order of DMF (91.0%) < ethanol (92.3%) ∼ DMSO (92.7%) < toluene (96.1%) < MIBK (99.7%) under HPMoV/meso-SiO_2_(18-f) catalyst, while the DFF yields followed the range of DMF (31.6%) < MIBK (49.0%) ethanol (68.6%) < toluene (77.5%) < DMSO (89.2%). Generally speaking, the selectivity of DFF depended on the polarity of solvents using HPMoV/meso-SiO_2_ catalyst and strong polarity and high boiling points were beneficial for 5-HMF conversion. The effects of organic solvents on the oxidation of 5-HMF were complicated, which might be due to the properties of solvents including polarity, dielectric constant, steric hindrance, and acid–base properties. Our results only provided the brief solvent effect on HMF oxidation; more in-depth investigation needs to be performed in the future. Nevertheless, the strong polarity and high boiling point solvent DMSO was found to be the best solvent for the transformation, in comparison to DMF, MIBK, toluene, and ethanol. Therefore, DMSO as the solvent was a suitable medium for oxidation of 5-HMF.

The influence of oxygen pressure was on DMSO with 80 mg of catalyst was investigated. The obtained results showed that higher oxygen pressure could enhance 5-HMF conversion, which enhanced oxygen pressure from 0.5 to 1.0 MPa, giving increased 5-HMF conversions from 86.3 to 92.7% and increased DFF yields from 80.5 to 89.2%, respectively. However, further increasing oxygen pressure to 1.5 MPa only enhanced HMF conversion to 97.0% but decreased yield of DFF, which was attributed to over-oxidation. Herein, the most suitable oxygen pressure was 1.0 MPa for oxidation of 5-HMF to DFF.

The influence of catalyst usages was investigated, which indicated that a lower conversion of 5-HMF with undesirable yield of DFF was obtain at 8 h reaction using lower usage of catalyst (Fig. 12b). Further increasing the usage of catalyst, DFF yield smoothly increased to the optimized yield of 89.2% with 80 mg catalyst. In contrast, increasing the amount of catalyst gave a high conversion of 5-HMF, but a sharply reduced yield of DFF owing to the subsequent oxidation of DFF to FDCA. Fig. 12c shows the influence of temperature on oxidation of 5-HMF to DFF catalyzed by HPMoV/meso-SiO_2_(18-f). Increasing the temperature promoted the aerobic oxidation of 5-HMF to DFF, while DFF yields sharply increased from 11.6% at 80 °C to 89.2% at 120 °C under the investigated conditions. A further enhancement of the reaction temperature to 130 °C resulted in a steep decrease in the DFF yield to 61.2%.

### 3.5 Reusability of HPMoV/SiO_2_ mesoporous nanofibers

**Fig. 13:**
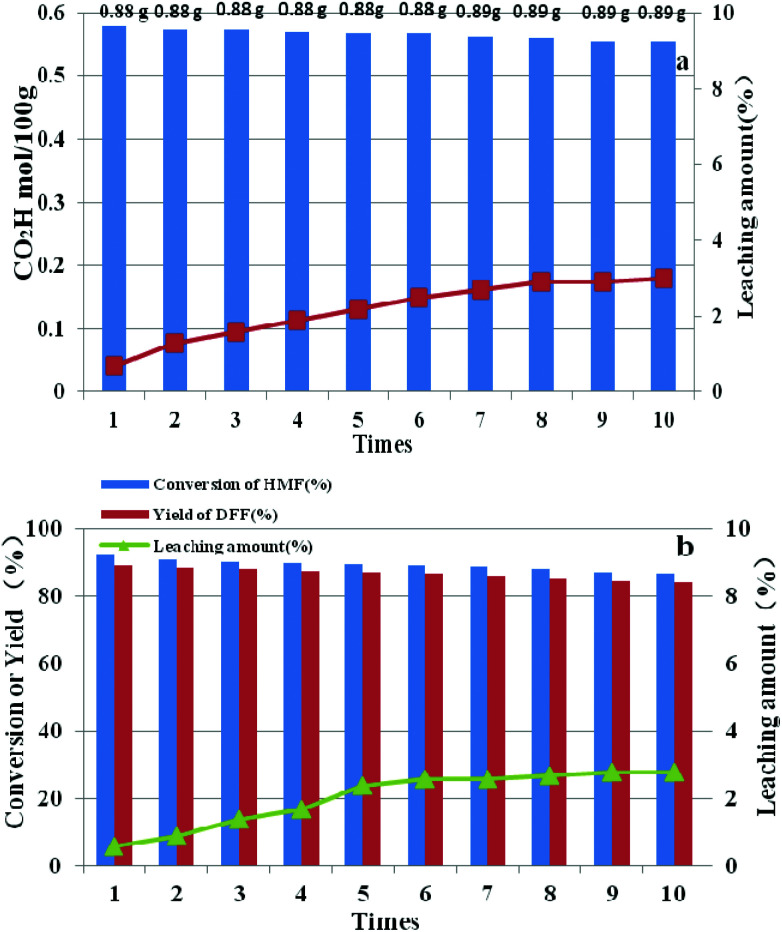
Reusability and leaching amount of HPMoV/SiO_2_(18-f) in oxidation of starch (a) and 5-HMF (b).

After each run, HPMoV/meso-SiO_2_(18-f) was separated from the reaction mixture by simple centrifugation and was reused by simply washing with H_2_O and drying at 80 °C. It could be seen that the carboxyl content for starch oxidation and DFF yields for 5-HMF oxidation showed only a slight decrease after ten cycles (Fig. 13). To investigate the potential leaching of H_5_PMo_10_V_2_O_40_ by silica, the reaction mixtures were studied by UV-vis spectroscopy after the catalytic experiment. The spectra did not exhibit any characteristic peaks of H_5_PMo_10_V_2_O_40_ in the range of 200–400 nm, indicating that no leaching of POMs took place under our reaction conditions (Fig. S8†) no matter whether the reaction occurred in the presence of O_2_ or H_2_O_2_. Therefore, the minor loss of catalytic activity of HPMoV/meso-SiO_2_(18-f) could be explained by the operation loss during recycling. The total loss of H_5_PMo_10_V_2_O_40_ were only 3.0% and 2.8%, respectively, corresponding to two reactions being tested by ICP, in which the loading amount of H_5_PMo_10_V_2_O_40_ on silica did not change during the reaction. The leaching test for HPMoV/meso-SiO_2_(18-f) was also performed to determine the leaching of H_5_PMo_10_V_2_O_40_ from silica. For starch oxidation, the catalyst was separated from the mixture by filtration after reaction for 2 h (0.35 mol per 100 g carboxyl content), and then the residual mixture further reacted for 2 h under the same conditions. For 5-HMF oxidation, the catalyst was separated by filtration after 1 h (15.2% 5-HMF conversion, 9.7% DFF yield), and then the filtrate was continually reacted for 1 h at the same conditions. After that, 0.1 mol per 100 g carboxyl content and 1.4% of 5-HMF conversion were achieved, which proved that negligible H_5_PMo_10_V_2_O_40_ dropped from the solid silica into the mixture during two reactions.

The IR spectrum of the used HPMoV/meso-SiO_2_(18-f) did not significantly differ from that of the fresh one, indicating that the structure of the heteropolyanion remained intact (Fig. S2†), while ^31^P-MAS NMR (Fig. S9†) and nitrogen adsorption analysis (Fig. S10†) also determined the structure, surface area and porosity were kept during oxidation reactions no matter whether using H_2_O_2_ or O_2_ as the oxidant.

The TEM of HPMoV/meso-SiO_2_(18-f) showed that the mesoporous structure did not change during the oxidation reaction (Fig. S11†). Meanwhile, the SEM images of the hybrid material (Fig. S12†) showed less changes in length of nanofibers during eight cycles while only the length of nanofibers was shortened after nine cycles under stirring. Therefore, it confirmed that the solid catalysts were not damaged and retained nanofiber morphology with the mesoporous structure in the oxidation reactions.

## 4. Conclusion

New POM solid catalyst HPMoV/SiO_2_ nanofibers with a mesoporous structure were successful synthesized *via* combined surfactant-directed pore formation and electrospinning techniques. This method provided a facile approach to fabricate mesoporous structural POM catalysts with unique physical properties such as high surface area, one-dimensional nanofiber morphology and excellent stability, which permitted HPMoV/SiO_2_ nanofibers to present higher efficiency in oxidation of starch by H_2_O_2_ and aerobic oxidation of HMF as well giving as high as 0.58 mol per 100 g carboxyl contents and 89.2% yield of DFF. One-dimensional nanofiber morphology and surrounded by silica support assisted POMs exhibited higher activity than homogeneous H_5_PMo_10_V_2_O_40_ and HPMoV/SiO_2_ without nanofiber morphology, which was due to the adsorption of more O_2_ and the limited decomposition of H_2_O_2_. HPMoV/SiO_2_ mesoporous nanofibers could be separated easily through filtration and could be reused for at least ten times without obvious decrease in catalytic activity in comparison with that of HPMoV/SiO_2_ mesoporous powder. Moreover, the oxidative starch and DFF were produced with highest TOF among the reported POM-based catalysts, indicating the availability for HPMoV/SiO_2_ mesoporous nanofibers in biomass conversion.

## Conflicts of interest

There are no conflicts to declare.

## Supplementary Material

RA-008-C7RA12842H-s001
