# Supplementary material for: Fabrication of mesoporous POMs/SiO2 nanofibers through electrospinning for oxidative conversion of biomass by H2O2 and oxygen
Source: RSC Adv. 2018 Jan 17;8(7):3499–511. doi: 10.1039/c7ra12842h (PMC9077668; doi:10.1039/c7ra12842h)
Supplement: RA-008-C7RA12842H-s001 [file RA-008-C7RA12842H-s001.pdf]

Electronic Supplementary Material (ESI) for RSC Advances.
This journal is © The Royal Society of Chemistry 2018

Supporting information

Fabrication of mesoporous POMs/SiO_2 nanofibers through electrospinning in oxidative conversion of biomass by H_2O_2 and oxygen

Siqi Yan, Yue Li, Peili Li, Ting Jia, Shengtian Wang, Xiaohong Wang*

Key Lab of Polyoxometalate Science of Ministry of Education, Faculty of Chemistry, Northeast Normal University, Changchun 130024, P. R. China

Fax: (+86)431-85099759; Tel: (86)431- 85099667; E-mail: wangxh665@nenu.edu.cn.

Postal address: Northeast Normal University, 5268 Renmin Street, Changchun, Jilin Province, 130024, P. R. China

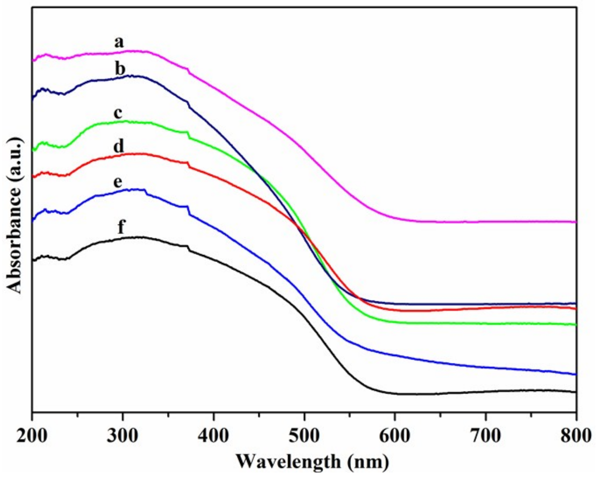

Fig. S1 The DR-UV-vis spectrum of HPMoV/meso-SiO_2(7-f) (a), HPMoV/meso-SiO_2(14-f) (b), HPMoV/meso-SiO_2(18-f) (c), HPMoV/meso-SiO_2(28.8-f) (d), HPMoV/meso-SiO_2(35-f) (e), and $H_5PMo_{10}V_2O_{40}$ (f).

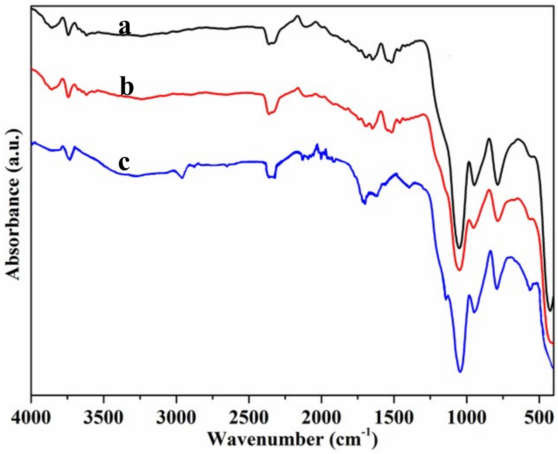

Fig. S2 IR spectra of HPMoV/meso-SiO_2(18-f) before reaction (a), after reaction (b), and absorbing starch (c)

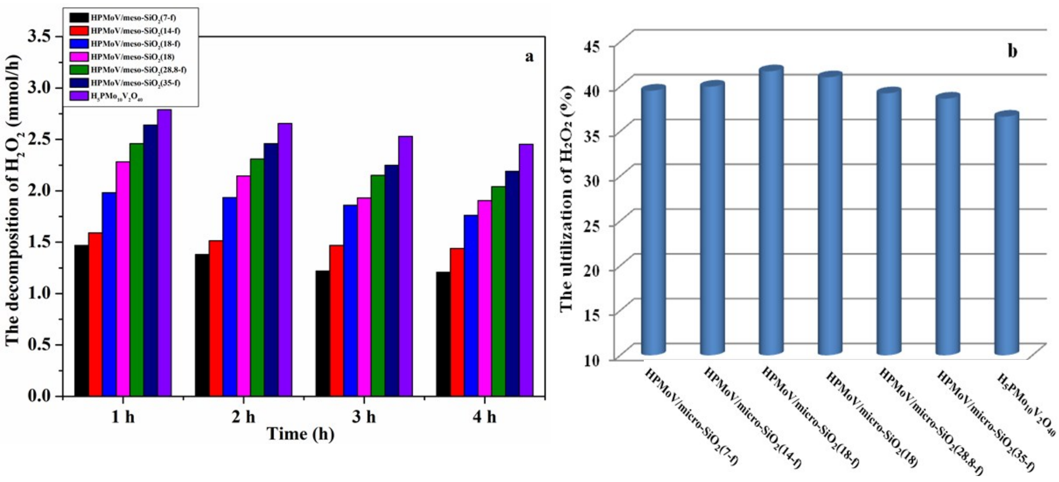

Fig. S3 The decomposition (a) and utilization (b) of H_2O_2 by various catalysts.

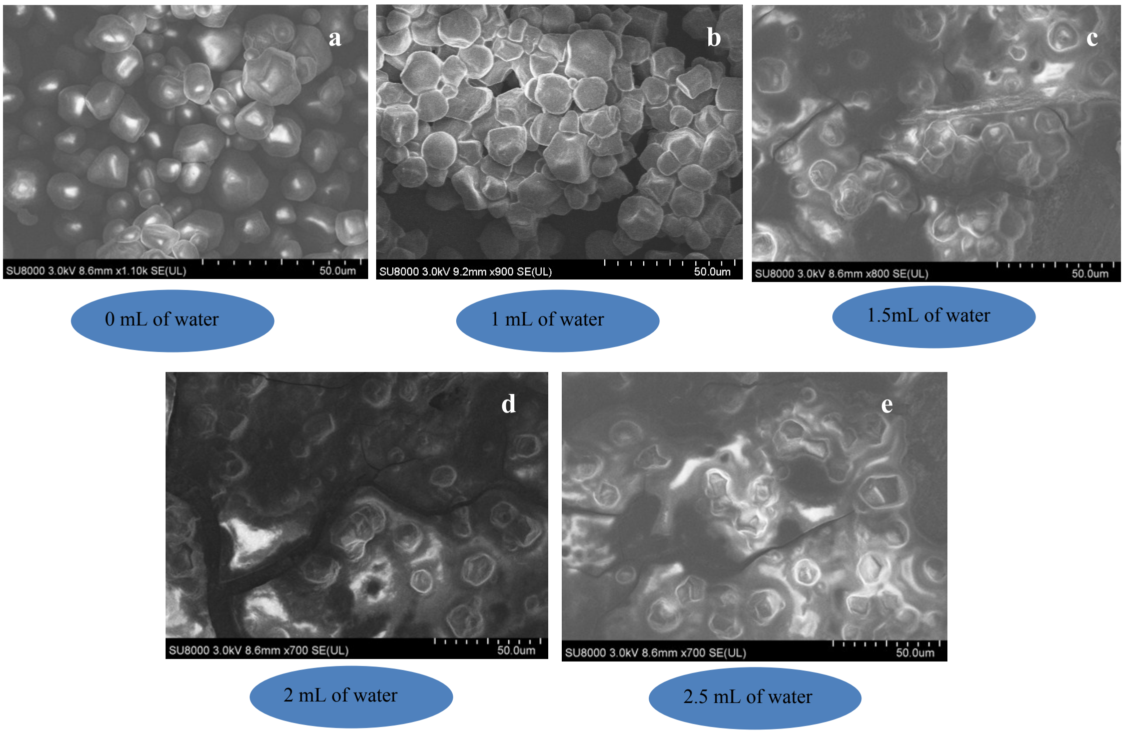

Fig. S4 The SEM images of starch with different water contents.

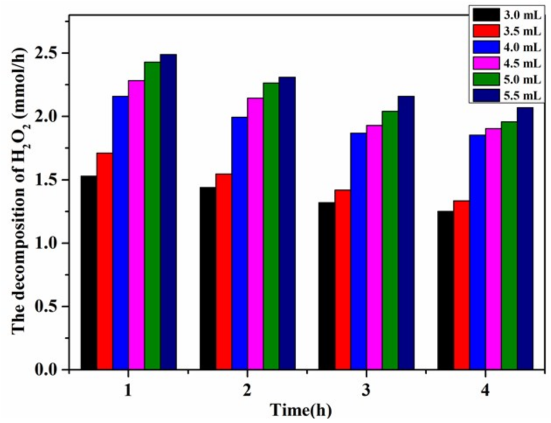

Fig. S5 The decomposition of H_2O_2 as using different usage of H_2O_2 by HPMoV/meso-SiO$_2$(18-f).

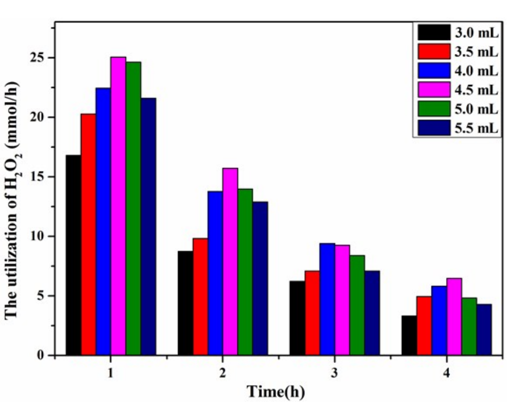

Fig. S6 The utilization of H_2O_2 in using different usage of H_2O_2 by HPMoV/meso-SiO_2(18-f)

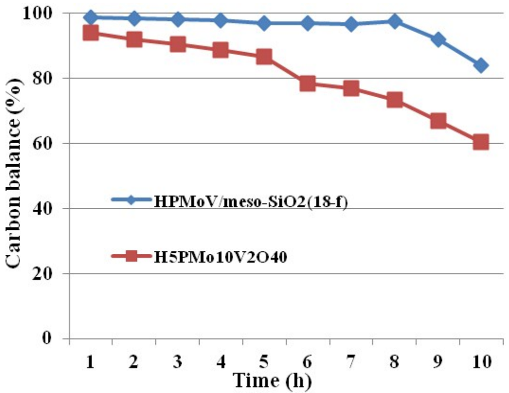

Fig. S7 The carbon balance for $H_5PMo_{10}V_2O_{40}$ and HPMoV/meso-SiO_2(18-f) for oxidation of 5-HMF with different time

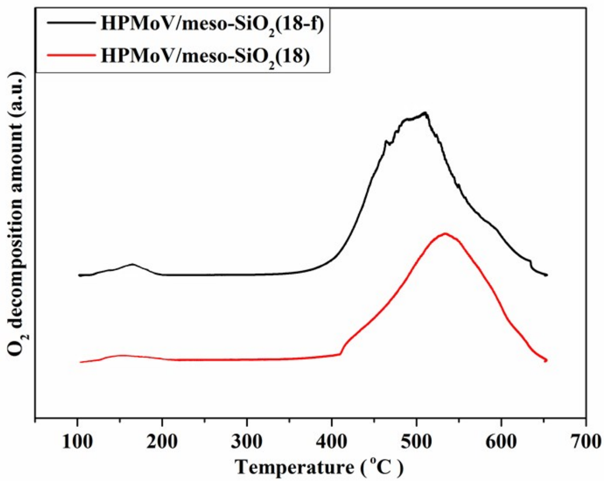

Fig. S8 O_2-TPD profiles of the HPMoV/meso-SiO_2(18-f) and HPMoV/meso-SiO_2(18).

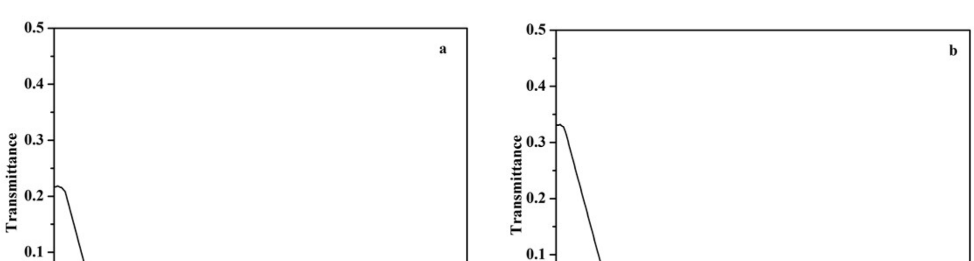

Fig. S9 Uv-Vis spectra of the reaction mixture obtained after starch oxidation (a) and 5-HMF conversion (b).

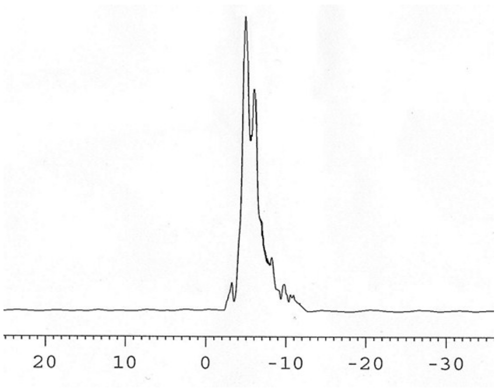

Fig. S10 The ^{31}P MAS NMR spectra of HPMoV/meso-SiO_2(18-f) after reaction

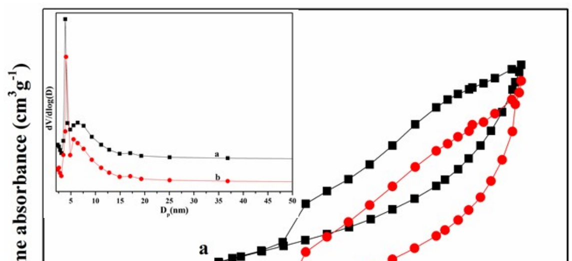

Fig. S11 Nitrogen adsorption–desorption isotherms and pore size distribution of HPMoV/meso-SiO_2(18-f) before reaction (a) and after reaction (b).

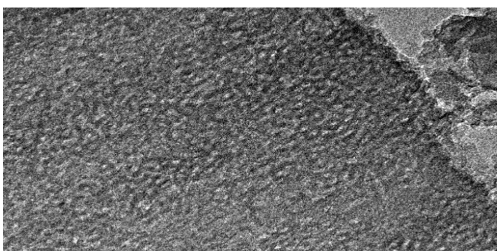

Fig. S12 TEM image of HPMoV/meso-SiO_2(18-f) after reaction

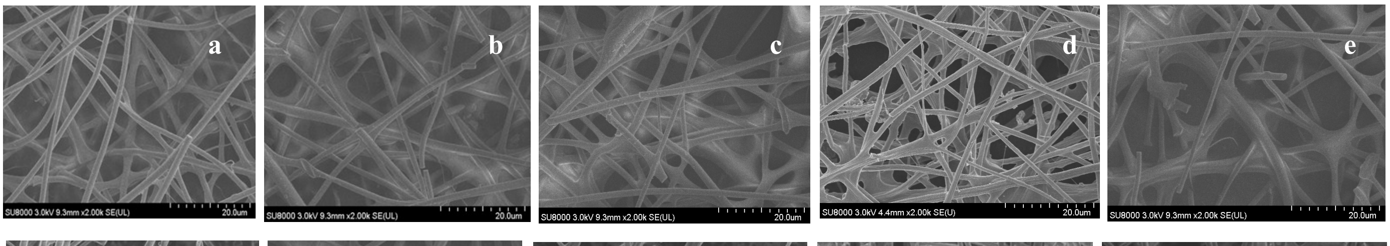

Fig. S13 SEM images of HPMoV/meso-SiO_2(18-f) after reaction for one to ten cycles (a-j)

Table S1 Oxidation of 5-HMF under various reaction conditions by different catalysts.

Catalysts	Reaction Condition	Oxidant	T (^oC)	Time (h)	Con. %	Yield of DFF %	Yield of FDCA%	Yield of MA%	Ref.
SBA-15-Biimidazole-Ru	0.5 mol 5-HMF<br>8 mL 4-chlorotoluene	O_2<br>20 mL/min	110	11	96.9	88.7	_	_	1
$ZnFe_{1.65}Ru_{0.35}O_4$	0.5 mol 5-HMF, 3 mL DMF	O_2<br>20 mL/min	110	4	94	82.2	3.4	_	2
Fe_3O_4@SiO_2-NH_2-Ru(III)	100 mg 5-HMF, 7 mL DMSO	O_2 balloon	120	4	99.3	86.4	_	_	3
Ru/HT	1mol 5-HMF, 3 mL DMF	O_2<br>20 mL/min	120	6	100	92	_	_	4
$Co_{6.6}Ce_{3.3}Ru_{1.1}$	1mol 5-HMF, 7 mL MIBK	O_2<br>20 mL/min	120	12	96.5	82.6	8.9	_	5
Ru/CTF	5-HMF/metal molar ratio 40:1<br>15 mL MTBE	Air<br>20 bar	80	1	86.3	63.6	_	_	6
γ-Fe_2O_3@HAP-Ru	100 mg 5-HMF<br>7 mL 4-chlorotoluene	O_2<br>0.1 MPa	90	4	100	89.1	_	_	7
Ru/CTF	1mmol 5-HMF, 15 mL H_2O	Air<br>20 bar	140	3	100	0.1	77.6	_	8
Ru/C	1 mmol 5-HMF, 10 mL H_2O	O_2<br>0.2 MPa	120	5	100	_	95	_	9
Ru/$MnCo_2O_4$	2 mmol 5-HMF, 20 mL H_2O	Air<br>0.7 MPa	120	10	100	_	99.1	_	10
Ag-OMS-2	315 mg 5-HMF<br>40 mL isopropyl alcohol	Air<br>15 atm	165	6	99	99	_	_	11

Au/TiO_2	Mole ratio NaOH:5-HMF=2<br>5 mL H_2O	O_2<br>2 MPa	23	6	100	_	79	_	12
Au/HT	1 mmol 5-HMF, 10 mL H_2O<br>1 mmol $NaHCO_3$	O_2 bubbling	95	7	100	_	99	_	13
Au/HT	1mmol 5-HMF, 6 mL H_2O	O_2<br>50 mL/min	90	7	99	_	99	_	14
Au/HY	0.317 g 5-HMF, 4.6 g H_2O<br>0.4 g NaOH	O_2<br>0.3 MPa	60	6	>99	_	>99	_	15
Au-CeO_2	2 mmol 5-HMF, NaOH/HMF=4<br>20 mL H_2O	Air<br>10 bar	65	8	100	_	>99	_	16
Au/TiO_2	1mmol 5-HMF, 10 mL H_2O	O_2<br>20 bar	30	18	99	_	71	_	17
Au_8-Pd_2/AC	0.15 M 5-HMF, 2 eq NaOH<br>5-HMF/metal=200 mol/mol	O_2<br>3 bar	60	4	99	_	99	_	18
Au-Pd/CNT	0.5 mmol 5-HMF, 20 mL H_2O	O_2<br>0.5 MPa	100	12	100	_	94	_	19
Au-Pd/ZOC	0.5 mmol 5-HMF, 10 mL H_2O<br>5-HMF/(Au+Pd)=100	O_2<br>3 bar	80	4	>99	_	>99	_	20
Pd/HT-5	0.4 mmol 5-HMF, 10 mL H_2O	O_2<br>100mL/min	100	8	100	_	99	_	21
C-Fe_3O_4-Pd	0.4 mmol 5-HMF, 8 mL H_2O<br>K_2CO_3/5-HMF = 2	O_2<br>30 mL/min	80	4	98.2	_	91.8	_	22
$Ni_{0.9}Pd_{0.1}$	2 mmol 5-HMF, 10 mL H_2O<br>5-HMF/$NaCO_3$=1	O_2<br>1.0 MPa	80	4	>99	_	86	_	23
Fe_3O_4@C@Pt	15 mg5-HMF, 0.2 mmol	O_2	90	4	100	[illegible]	100	[illegible]	24

Pt/TiO_2	Na_2CO_3<br>2 g 5-HMF, $NaHCO_3$/5-HMF=2	100mL/min<br>Air 0.1 MPa	100	6	>99.9	_	>99.9	_	25
Pt/C-O-Mg	0.5 mmol 5-HMF, 10 mL H_2O	O_2<br>1.0 MPa	110	12	100	_	97	_	26
$Pt_{imp}Bi/CL_{35}$	0.1 M 5-HMF<br>Mole ratio $NaHCO_3$/5-HMF = 4	Air<br>50 bar	100	3	100	_	99	_	27
Pt/CNT	0.5 mmol 5-HMF, 20 ml H_2O<br>5-HMF/metal(molar ratio,100:1)	O_2<br>0.5 MPa	90	14	100	_	98	_	28
FeCo/C	1mol 5-HMF<br>1 mmol Na_2CO_3, 2 mL Toluene	O_2<br>1.0 MPa	100	6	99	99	_	_	29
Co-P/Co-P	50 mM 5-HMF, 1.0 M KOH<br>current density of 20 mA/cm^2	_	25	6	100	_	90	_	30
Co/Mn/Br	13.2 mmol 5-HMF<br>H_2O/HOAc = 7/93<br>(5 mL HOAc)	N_2/O_2 = 1/1<br>(mol/mol)	160	0.5	100	_	78.1	_	31
Co/Mn/Br	2.2-2.3 mmol 5-HMF, 2.5 mL<br>HOAc	O_2<br>0.76 MPa	180	1/6	>99	_	92.9	_	32
$Bi(NO_3)_3\cdot 5H_2O$, cellulose-<br>Cu-NP	126 mg 5-HMF, 10 mL MeCN	Air	80	2	96.5	82	_	_	33
$Co(OAc)_2$/<br>$Zn(OAc)_2/Br^-$	240 mM 5-HMF<br>HOAc	O_2<br>10 bar	90	6	90	_	60	_	34
CuI	1.26 g 5-HMF, 15 mL DMSO	O_2<br>0.3 MPa	130	10	93.2	92.3	_	_	35
$Ni_3(BTP)_2$	1.8 mmol 5-HMF, 25 mL water	O_2<br>30 bar	120	24	100	99	_	_	36
V-g-C_3N_4	1mol 5-HMF, 2 mL DMSO	O_2	130	6	99	82			37

		0.1 MPa								
Polyaniline-$VO(acac)_2$	100 mg 5-HMF<br>8 mL 4-chlorotoluene	O_2<br>20 mL/min	110	12	99.2	86.2	_	_		38
$VO(acac)_2$	2.5 mmol 5-HMF<br>5 mL acetonitrile,	O_2<br>1.0 MPa	90	4	99	14	_	52		39
$C_{14}VOHPO_4$	0.1 M 5-HMF, 5 mL Toluene	O_2<br>1 atm	110	6	99	83	_	_		40
α-CuV_2O_6	1mol 5-HMF, 4 mL DMSO	O_2<br>1 bar	130	3	100	99.9	_	_		41
$Cu(NO_3)_2/VOSO_4$	10 mmol 5-HMF, 5 mL acetonitrile	O_2<br>0.1 MPa	80	5	99	99	_	_		42
VO_2-PANI/CNT	1mol 5-HMF, 2 mL DMSO	O_2<br>1.0 MPa	120	11	>99	96	_	_		43
SBA-NH_2-VO^{2+}<br>SBA-NH_2-Cu^{2+}	100 mg HMF<br>7 mL 4-chlorotoluene	O_2<br>0.28 MPa	110	12	98.8	62.7	_	_		44
VO-NH_2-GO	2.0 mmol 5-HMF<br>10 mL acetic acid	O_2<br>20 bar	90	4	99.8	2.4	_	95.3		45
$VOHPO_4$	75 mmol 5-HMF, 50 mL DMSO	Air	150	13	100	61	_	_		46
V_2O_5/H-beta	100 mg 5-HMF, 5 mL DMSO	O_2<br>30 mL/min	125	6	84	82	11.7	_		47
V_2O_5/ceramic	63 mg 5-HMF, 4 mL DMSO	O_2<br>40 mL/min	140	5	100	87.5	_	_		48
V_2O_5	2.0 mmol 5-HMF<br>10 mL acetic acid	O_2<br>10 bar	100	4	99	_	_	75		49
Fe_3O_4@SiO_2-TEMPO	1mol 5-HMF, 75 mg HOAc<br>2 mL Toluene	_	50	18	100	99	_	_		50

Fe_3O_4-CoO_x	70 mg 5-HMF, 4 mL DMSO	t-BuOOH 0.5 mL	80	15	97.2	_	68.6	_	51
Fe_3O_4/Mn_3O_4	126 mg 5-HMF, 7 mL DMF	O_2 20 mL/min	120	4	99.8	82.1	_	_	52
MnO_2	0.2 mmol 5-HMF, 5 mL H_2O 0.6 mmol $NaHCO_3$	O_2 1.0 MPa	100	24	≥99	_	91	_	53
K-OMS-2	100 mg 5-HMF, 3 mL DMSO	O_2 10 mL/min	110	6	99	99	_	_	54
CC-SO_3H-NH_2	200 mg 5-HMF, 2 mL DMSO	O_2 20 mL/min	140	9	85	85	_	_	55
N-doped graphene NG-800	1mol 5-HMF, 30 mL acetonitril	1 mmol TEMPO	100	6	100	99.5	_	_	56
NNC-900	0.63 mmol 5-HMF, 10 mL H_2O HMF/K_2CO_3= 3	O_2 100mL/min	80	48	100	_	80	_	57
GO + catalase + HRP	30 mM 5-HMF 50 mM, 2 mL phosphate buffer	alcohol oxidases	25	96	91	91	_	_	58
GO	2 mmol 5-HMF, 4 mL DMSO	O_2 20 mL/min	140	24	100	90	_	_	59
GO	1mol 5-HMF, 30 mL acetonitrile 1mol TEMPO	_	100	18	100	99.6	_	_	60
GO_{ase} $M_{3\text{-}5}$+PaoABC	0.3 mmol 5-HMF,0.3 mL MeCN 400 mM, 1.09 mL potassium phosphate buffer	Air	37	10	100	_	86.9	_	61
FAD-dependent enzyme	2 mM 5-HMF 100 mM potassium phosphate buffer	_	25	24	100	_	95	_	62

NaBr	1mol 5-HMF, 2 mL DMSO	_	150	18	100	85	_	_	63
$NaNO_2$	100 mg 5-HMF<br>4 mL Trifluoroacetic acid	_	25	1	98.3	89.8	_	_	64
$H_5PMo_{10}V_2O_{40}$	2.4 mmol 5-HMF<br>2 mL acetonitrile<br>1.3 mL acetic acid	O_2<br>10 atm	90	8	100	_	_	64	65
$[EMIM]_4Mo_8O_{26}$	9 mmol 5-HMF, 18 mmol NaOH<br>20 mL H_2O	25 ml 6%<br>H_2O_2	100	2	96.2	_	92.7	_	66
$Cs_3HPMo_{11}VO_{40}$	1mol 5-HMF, 2 mL DMSO	O_2<br>0.8 MPa	110	6	99	99	_	_	67
PMA-MIL-101	0.5 mol 5-HMF, 5 mL DMSO	O_2<br>20 mL/min	140	20	90.8	88.2	_	_	68

Table S2 Oxidation of starch by hydrogen peroxide in the literature

**Catalysts**	**T (°C)**	**Time (h)**	**H_2O_2 mL**	**CO_2H contents mol/100g**	**Ref.**
$FeSO_4$	40	24	6.8	0.16	69
Na_2WO_4	90	2.6	3.75	0.249	70
$FeSO_4$	60	24	2	0.61	71
FePcS	60	14	5	4.0	72
$Ag_{3.5}(NH_4)_{1.5}PMo_{10}V_2O_{40}$	80	10	4.0	0.62	73
$[C_{16}H_{33}N(CH_3)_3]_5PV_2Mo_{10}O_{40}$	70	10	4.5	0.59	74
$(NH_4)_5H_6PV_8Mo_4O_{40}$	70	10	2.5	0.648	75
$Cs_3H_2PMo_{10}V_2O_{40}$	70	10	4.5	0.59	76
$[(CH_3)_3NCH_2CH_2OH]_5PV_2Mo_{10}O_{40}$	70	10	4.5	0.59	77

Table S3 Different catalysts for uptaking amount of oxygen.

**Cat.**	micro-SiO_2	meso-SiO_2	micro-SiO_2-f	meso-SiO_2-f	HPMoV/micro-SiO_2(18)	HPMoV/meso-SiO_2(18)
**Amount of oxygen (mol/g)**	5.06×10^{-6}	8.86×10^{-5}	1.05×10^{-5}	1.16×10^{-4}	3.79×10^{-6}	4.30×10^{-5}
**Cat.**	HPMoV/meso-SiO_2（7-f）	HPMoV/meso-SiO_2（14-f）	HPMoV/meso-SiO_2（18-f）	HPMoV/meso-SiO_2（28.8-f）	HPMoV/meso-SiO_2（35-f）	$H_5PMo_{10}V_2O_{40}$
**Amount of oxygen (mol/g)**	8.10×10^{-5}	6.83×10^{-5}	5.60×10^{-5}	3.79×10^{-5}	7.59×10^{-6}	5.06×10^{-7}

References

[1] F. Wang, L. Jiang, J. M. Wang and Z. H. Zhang, *Energy fuels,* 2016, **30**, 5885-5892.

[2] Z. Z. Yang, W. Qi, R. X. Su and Z. M. He, *Energy fuels*, 2017, **31**, 533-541.

[3] S. G. Wang, Z. H. Zhang, B. Liu and J. L. Li, *Ind. Eng. Chem. Res*., 2014, **53**, 5820-5827.

[4] A. Takagaki, M. Takahashi, S. Nishimura and K. Ebitani, *ACS Catal*., 2011, **1**, 1562-1565.

[5] Y. M. Wang, B. Liu, K. C. Huang, Z. H. Zhang, *Ind. Eng. Chem. Res.*, 2014, **53**, 1313-1319.

[6] J. Artz, S. Mallmann and R. Palkovits, *ChemSusChem.*, 2015, **8**, 672-679.

[7] Z. H. Zhang, Z. L. Yuan, D. G. Tang, Y. S. Ren, K. L. Lv and B. Liu, *ChemSusChem.*, 2014, **7**, 3496-3504.

[8] J. Artz, R. Palkovits, *ChemSusChem.*, 2015, **8**, 3832-3838.

[9] G. S. Yi, S. P. Teong and Y. G. Zhang, Green Chem., 2016, **18**, 979-983.

[10] D. K. Mishra, H. J. Lee, J. S. Kim, H. S. Lee, J. K. Cho, Y. W. Suh, Y. J. Yi and Y. J. Kim, *Green Chem*., 2017, **19**, 1619-1623

[11] G. D. Yadav, R. V. Sharma, *Appl. Catal., B: Environ.*, 2014, **147**, 293-301

[12] S. E. Davis, B. N. Zope, R. J. Davis, *Green Chem*., 2012, **14**, 143-147

[13] G. S. Yi, S. P. Teong, X. K. Li and Y. G. Zhang*, ChemSusChem.*, 2014, **7**, 2131-2135

[14] N. K. Gupta, S. Nishimura, A. Takagaki and K. Ebitani, *Green Chem.,* 2011, **13**, 824-827

[15] J. Y. Cai, H. Ma, J. Y. Zhang, Q. Song, Z. T. Du, Y. Z. Huang, J. Xu, *Chem. Eur. J*., 2013, **19**, 14215-14223.

[16] O. Casanova, S. Iborra, A. Corma, *ChemSusChem.,* 2009, **2**, 1138-1144.

[17] Y. Y. Gorbanev, S. K. Klitgaard, J. M. Woodley, C. H. Christensen and A. Riisager, *ChemSusChem*., 2009, **2**, 672-675.

[18] A. Villa, M. Schiavoni, S. Campisi, G. M. Veith, L. Prati, *ChemSusChem.,* 2013, **6**, 609-612.

[19] X. Y. Wan, C. M. Zhou, J. S. Chen, W. P. Deng, Q. H. Zhang, Y. H. Yang and Y. Wang, *ACS Catal*., 2014, **4**, 2175-2185.

[20] Z. Y. Gui, W. R. Cao, S. Saravanamurugan, A. Riisager, L. F. Chen and Z. W. Qi, *ChemCatChem.,* 2016, **8**, 3636-3643.

[21] Y. B. Wang, K. Yu, D. Lei, W. Si, Y. J. Feng, L. L. Lou, S. X. Liu, *ACS Sustain. Chem. Eng*., 2016, **4**, 4752-4761.

[22] N. Mei, B. Liu, J. D. Zheng, K. L. Lv, D. G. Tang and Z. H. Zhang, *Catal. Sci., Technol.*, 2015, **5**, 3194-3202.

[23] K. Gupta, R. K. Rai, A. D. Dwivedi and S. K. Singh, *ChemCatChem.,* 2017, **9**, 2760-2767.

[24] Y. W. Zhang, Z. M. Xue, J. F. Wang, X. H. Zhao, Y. H. Deng, W. C. Zhao and T. C. Mu, *RSC Adv*., 2016, **6**, 51229-51237.

[25] H. A. Rass, N. Essayem and M. Besson, *ChemSusChem.,* 2015, **8**, 1206-1217.

[26] X. W. Han, L. Geng, Y. Guo, R. Jia, X. H. Liu, Y. G. Zhang and Y. Q. Wang, *Green Chem*., 2016, **18**, 1597-1604.
[27] H. A. Rass, N. Essayem and M. Besson, *Green Chem.*, 2013, **15**, 2240-2251.
[28] C. M. Zhou, W. P. Deng, X. Y. Wan, Q. H. Zhang, Y. H. Yang and Y. Wang, *ChemCatChem.*, 2015, **7**, 2853-2863.
[29] R. Q. Fang, R. Luque and Y. W. Li, *Green Chem.*, 2016, **18**, 3152-3157.
[30] N. Jiang, B. You, R. Boonstra, I. M. T. Rodriguez and Y. J. Sun, *ACS Energy Lett.*, 2016, **1**, 386-390.
[31] X. B. Zuo, P. Venkitasubramanian, D. H. Busch and B. Subramaniam, *ACS Sustain. Chem. Eng.,* 2016, **4**, 3659-3668.
[32] X. B. Zuo, A. S. Chaudhari, K. Snavely and F. H. Niu, *AIChE J*., 2017, **63**, 162-171.
[33] D. Baruah, F. L. Hussain, M. Suri, U. P. Saikia, P. Sengupta, D. K. Dutta and D. Konwar, *Catal. Commun.*, 201, 6, **77**, 9-12.
[34] B. Saha, S. Duttaa and M. M. Abu-Omar, *Catal. Sci. Technol*., 2012, **2**, 79-81.
[35] X. L. Tong, Y. F. Sun, X. Q. Bai and Y. D. Li, *RSC Adv*., 2014, **4**, 44307-44311.
[36] C. Lucarelli, S. Galli, A. Maspero, A. Cimino, C. Bandinelli, A. Lolli, J. V. Ochoa, A. Vaccari, F. Cavani and S. Albonetti, *J. Phys. Chem. C*, 2016, **120**, 15310-15321.
[37] J. Z. Chen, Y. Y. Guo, J. Y. Chen, L. Song and L. M. Chen, *ChemCatChem*., 2014, **6**, 3174-3181.
[38] F. H. Xu, Z. H. Zhang, *ChemCatChem.,* 2015, **7**, 1470-1477.
[39] Z. T. Du, J. P. Ma, F. Wang, J. X. Liu and J. Xu, *Green Chem*., 2011, **13**, 554-557.
[40] F. L. Grasset, B. Katryniok, S. Paul, V. N. Rataj, M. P. Titus, J. M. Clacens, F. D. Campof and F. Dumeignil, *RSC Adv*., 2013, **3**, 9942-9948.
[41] W. Hou, Q. Wang, Z. J. Guo, J. Li, Y. Zhou and J. Wang, *Catal. Sci. Technol*., 2017, **7**, 1006-1016.
[42] J. P. Ma, Z. T. Du, J. Xu, Q. H. Chu and Y. Pang, *ChemSusChem.,* 2011, **4**, 51-54.
[43] Y. Y. Guo, J. Z. Chen, *ChemPlusChem*., 2015, **80**, 1760-1768.
[44] L. F. Liao, Y. Liu, Z. Y. Li, J. P. Zhuang, Y. B. Zhou and S. Chen, *RSC Adv*., 2016, **6**, 94976-94988.
[45] G. Q. Lv, C. Y. Chen, B. Q. Lu, J. L. Li, Y. X. Yang, C. M. Chen, T. S. Deng, Y. L. Zhu and X. L. Hou, *RSC Adv*., 2016, **6**, 101277-101282.
[46] G. A. Halliday, R. J. Young, and V. V. Grushin, *Org. Lett.*, 2003, **5**, 2003-2005.
[47] I. Sádaba, Y. Y. Gorbanev, S. Kegnæs, S. S. R. Putluru, R. W. Berg and A. Riisager, *ChemCatChem.*, 2013, **5**, 284-293.
[48] M. Cui, R. L. Huang, W. Qi, R. X. Su, Z. M. He, *RSC Adv*., 2017, **7**, 7560-7566.
[49] X. K. Li, Y. G. Zhang, *Green Chem*., 2016, **18**, 643-647.
[50] B. Karimi, H. M. Mirzaei and E. Farhangi, *ChemCatChem.*, 2014, **6**, 758-762.

[51] S. G. Wang, Z. H. Zhang and B. Liu, *ACS Sustain. Chem. Eng*., 2015, **3**, 406-412.
[52] B. Liu, Z. H. Zhang, K. L. Lv, K. J. Deng and H. M. Duan, *Appl. Catal. A: Gen.*, 2014, **472**, 64-71.
[53] E. Hayashi, T. Komanoya, K. Kamata and M. Hara, *ChemSusChem,* 2017, **10**, 654-658.
[54] Z. Z. Yang, J. Deng, T. Pan, Q. X. Guo and Y. Fu, *Green Chem.*, 2012, **14**, 2986-2989.
[55] P. V. Rathod, S. D. Nale and V. H. Jadhav, *ACS Sustain. Chem. Eng.*, 2017, **5**, 701-707.
[56] G. Q. Lv, H. L. Wang, Y. X. Yang, X. Li, T. S. Deng, C. M. Chen, Y. L. Zhu and X. L. Hou, *Catal. Sci. Technol.*, 2016, **6**, 2377-2386.
[57] C.V. Nguyen, Y. T. Liao, T. C. Kang, J. E. Chen, T. Yoshikawa, Y. Nakasaka, T. Masudab and K. C. W. Wu, *Green Chem*., 2016, **18**, 5957-5961.
[58] Y. Z. Qin, Y. M. Li, M. H. Zong, H. Wu and N. Li, *Green Chem*., 2015, **17**, 3718-3722.
[59] G. Q. Lv, H. L. Wang, Y. X. Yang, X. Li, T. S. Deng, C. M. Chen, Y. L. Zhu and X. L. Hou, *Green Chem*., 2016, **18**, 2302-2307.
[60] G. Q. Lv, H. L. Wang, Y. X. Yang, T. S. Deng, C. M. Chen, Y. L. Zhu, X. L. Hou, *ACS Catal*., 2015, **5**, 5636-5646.
[61] S. M. McKenna, S. Leimkühler, S. Herter, N. J. Turner and A. J. Carnell, *Green Chem*., 2015, **17**, 3271-3275.
[62] W. P. Dijkman, D. E. Groothuis and M. W. Fraaije, *Angew. Chem. Int. Ed.*, 2014, 53, 6515-6518.
[63] C. Laugel, B. Estrine, J. L. Bras, N. Hoffmann, S. Marinkovic and J. Muzart, *ChemCatChem.*, 2014, **6**, 1195-1198.
[64] X. X. Liu, H. Ding, Q. Xu, W. Z. Zhong, D. L. Yin and S. P. Su, *J. Energ. Chem*., 2016, **25**, 117-121.
[65] J. H. Lan, J. C. Lin, Z. Q. Chen, G. C. Yin, *ACS Catal*., 2015, **5**, 2035-2041.
[66] S. Li, K. M. Su, Z. H. Li and B. W. Cheng, *Green Chem.,* 2016, **18**, 2122-2128.
[67] R. L. Liu, J. Z. Chen, L. M. Chen, Y. Y. Guo and J. W. Zhong, *ChemPlusChem.*, 2014, **79**, 1448-1454.
[68] J. Zhao, J. Anjali, Y. B. Yan and J. M. Lee, *ChemCatChem.*, 2017, **9**, 1187-1191.
[69]P. Parovuori, A. Hamunen, P. Forssell, K. Autio and K. Poutanen, *starch/starke,* 1995, **47**, 19-23.
[70]M. Floor, K. M. Schenk, A. P. G. Kieboom and H. V. Bekkum, *starch/starke* , 1989, 4**1**, 303-309.
[71]C. Aouf, D. Harakat, J. Muzart, B. Estrine, S. Marinkovic, C. Ernenwein, J. L. Bras, *ChemSusChem.,* 2010, **3**, 1200-1203.
[72]S. L. K. Sorokina, P. Gallezot, A. B. Sorokin, *Chem. Commun*, 2004, 2844-2845.
[73]X. L. Chen, H. Wang, J. Xu, M. X. Huo, Z. J. Jiang, X. H. Wang, *Catal. Today*, 2014, **234**, 264-270.
[74]H. Wang, X. L. Chen, L. L. Zhao, X. H. Wang, L. C. Wang, *Catal. Surv. Asia*, 2015, **19**, 123-128.
[75] H. Wang, Y. Poya, X. L. Chen, T. Jia, X. H. Wang, J.Y. Shi, *RSC Adv*., 2015, **5**, 45725-45730.
[76]X. L. Chen, Y. Liu, H. Wang, M. J. Yuan, X. H. Wang, Y. G. Chen, *RSC Adv*., 2014, **4**, 11232-11239.

[77]X. L. Chen, B. Souvanhthonga, H. Wang, H. W. Zheng, X. H. Wang, M. X. Huo, *Appl. Catal., B: Environ.*, 2013, **138-139**, 161-166.
